# Salvage Magnetic Resonance Imaging–guided Transurethral Ultrasound Ablation for Localized Radiorecurrent Prostate Cancer

**DOI:** 10.1016/j.euros.2024.11.001

**Published:** 2024-12-05

**Authors:** Mikael Anttinen, Pietari Mäkelä, Pertti Nurminen, Heikki Pärssinen, Simona Malaspina, Teija Sainio, Mikael Högerman, Pekka Taimen, Roberto Blanco Sequeiros, Peter J. Boström

**Affiliations:** a Department of Urology, University of Turku and Turku University Hospital, Turku, Finland; b Department of Diagnostic Radiology, University of Turku and Turku University Hospital, Turku, Finland; c Turku PET Centre, University of Turku and Turku University Hospital, Turku, Finland; d Department of Medical Physics, University of Turku and Turku University Hospital, Turku, Finland; e Institute of Biomedicine, University of Turku and Department of Pathology, Turku University Hospital, Turku, Finland

**Keywords:** Ablation therapy, Biochemical recurrence, Local recurrence, Magnetic resonance imaging–guided transurethral ultrasound ablation, Prostate cancer, Radiorecurrent prostate cancer, Salvage therapy, Transurethral ultrasound ablation, Ultrasound ablation

## Abstract

**Background and objective:**

Toxicity from local salvage therapy for radiorecurrent prostate cancer (PCa) remains a concern. This phase 2 study evaluates the outcomes of salvage magnetic resonance imaging (MRI)-guided transurethral ultrasound ablation (sTULSA).

**Methods:**

Men with biochemically relapsed, biopsy-proven PCa following definitive radiotherapy underwent whole- or partial-gland sTULSA (NCT03350529). Prostate-confined recurrence was confirmed by MRI and prostate-specific membrane antigen (PSMA) positron emission tomography (PET) computed tomography (CT). The primary endpoints were safety (Clavien-Dindo classification) and efficacy (prostate-specific antigen [PSA], PSMA PET-CT, and MRI-targeted biopsy at 12 mo). The secondary endpoints included functional and survival outcomes.

**Key findings and limitations:**

Thirty-nine patients underwent sTULSA (64% whole gland), with a median age of 73 yr (interquartile range [IQR]: 69–77) and PSA of 3.3 ng/ml (IQR: 2–6.2). Three patients had undergone prior salvage therapy, 16 were receiving hormonal therapy at enrollment, and 12 had a history of transurethral interventions. Eighteen patients had incidental urethral strictures on baseline cystoscopy. Over a median follow-up of 40 mo (IQR: 24–55), 56% experienced adverse events. Severe genitourinary toxicity (Clavien-Dindo ≥3 or hospitalization) occurred in 28%, including three patients with puboprostatic fistulas and two patients requiring cystectomy. Leak-free continence was maintained in 53%. At 12 mo, 89% showed no cancer in the targeted area, with a median PSA reduction of 95% (*p* < 0.001). Five-year metastasis-free, failure-free, and biochemical recurrence–free survival probabilities (95% confidence interval) were 97% (0.93–1.00), 70% (0.54–0.91), and 54% (0.31–0.93), respectively. Limitations included single-arm design and moderate sample size.

**Conclusions and clinical implications:**

It has been observed that sTULSA is effective for radiorecurrent PCa, although genitourinary toxicity remains a concern. Further studies should refine patient selection and treatment parameters to improve safety and tolerability.

**Patient summary:**

In this study, we examined a new treatment called magnetic resonance imaging–guided transurethral ultrasound ablation for prostate cancer that has returned after radiation therapy. We found that the treatment provided effective and lasting cancer control for most patients. However, a notable number of patients experienced significant genitourinary toxicity, including severe adverse effects affecting urinary function. Careful patient selection is crucial to minimize these adverse effects and ensure the best results.

## Take Home Message

Salvage magnetic resonance imaging–guided transurethral ultrasound ablation shows promising oncological control for localized radiorecurrent prostate cancer, although genitourinary toxicity remains a concern. Future studies should focus on refining its application and improving outcomes.

## 1 Introduction

Definitive radiotherapy (RT), particularly external beam radiotherapy (EBRT) combined with adjuvant androgen deprivation therapy (ADT), remains a cornerstone of treatment for localized prostate cancer (PCa), alongside surgery [1]. Advances in dose escalation, fractionation schemes, and imaging guidance have enhanced the efficacy and safety of RT [1].

Despite these advances, local recurrence remains a frequent challenge and can progress to metastatic or symptomatic disease if untreated [2], [3], [4], [5], [6]. Optimal management of local recurrence is unclear, largely due to the absence of large prospective trials. Conventional salvage treatments, such as radical prostatectomy (RP) [7], reirradiation with stereotactic body RT (SBRT) [8] or high-dose-rate brachytherapy (HDRb) [9], [10], [11], high-intensity focused ultrasound (HIFU) [12], and cryoablation [13], are associated with significant toxicity and limited efficacy [6]. Conventional imaging lacks sensitivity to detect micrometastases, leading to recurrence from undetected metastatic disease.

Advanced imaging modalities, such as multiparametric magnetic resonance imaging (mpMRI) and prostate-specific membrane antigen (PSMA) positron emission tomography (PET), have revolutionized lesion localization in recurrent PCa, improving patient selection by distinguishing between local and systemic disease and enhancing intraprostatic tumor detection and localization [1], [14], [15], [16]. This allows for more precise targeting of therapies, potentially improving clinical outcomes. Minimally invasive ablation techniques such as transrectal HIFU and transperineal cryoablation aim to preserve genitourinary (GU) function while providing outcomes comparable with radical treatment [16]. However, prostate size and tumor location limit patient selection, restricting treatment to specific regions within the prostate [17].

To address these limitations, MRI-guided transurethral ultrasound ablation (TULSA) has emerged as a versatile treatment option [15], [18], [19]. TULSA provides real-time MRI guidance and precise tissue ablation, allowing treatment of all prostate regions within a 3 cm radius from the urethra in a single session, making it suitable for personalized treatment of recurrent prostate tumors.

Our phase 1 study established the feasibility of salvage TULSA (sTULSA) for radiorecurrent PCa [20]. The current study expands on these findings, addressing the gap in prospective data by assessing oncological and safety outcomes in a larger cohort with extended follow-up.

## 2 Patients and methods

### 2.1 Study design

This prospective, investigator-initiated, single-arm, single-center phase 2 study (NCT03350529) was built on our phase 1 sTULSA findings [20]. Owing to promising 12-mo results, the cohort was expanded to 40 patients to further evaluate safety and efficacy, including the previously reported phase 1 patients (*n* = 11), whose longer-term outcomes are now reported. The protocol was approved by the ethics committee, and all participants provided written informed consent in accordance with the Declaration of Helsinki.

### 2.2 Patient eligibility and selection

Eligible participants were men with Phoenix biochemical recurrence (BCR), defined as a prostate-specific antigen (PSA) increase of 2 ng/ml above the post-RT nadir [21], with no history of distant metastasis. Within 3 mo before sTULSA, patients received pelvic 3 T mpMRI, whole-body fluorine-18 PSMA-1007 (18F-PSMA-1007) PET computed tomography (CT), and MRI-targeted biopsy to confirm prostate-confined recurrence **(**Supplementary material). Only MRI-positive patients were included, defined as having a Likert or Prostate Imaging for Recurrence Reporting (PI-RR) score of ≥3 for the lesion, depending on recruitment timing [22]. All MRI scans were interpreted by the same radiologists with over 7 yr of expertise in prostate MRI and ablation. Outpatient flexible cystoscopy ensured urethral patency for device insertion. The tumor’s peripheral edge had to be ≤3 cm from the urethra center on baseline MRI. The exclusion criteria included extraprostatic disease, MRI contraindications, metal objects in the pelvic area (except post-RT fiducial markers) [23], [24], and prostate calcifications or cysts larger than 1 cm within the target volume.

### 2.3 Description of the intervention

The treatment was administered using the TULSA-PRO system (Profound Medical Inc., Mississauga, Canada) [10]. A detailed description of the technology and treatment strategy is provided in the Supplementary material. Based on tumor characteristics and patient preference, patients received either whole-gland (WG) ablation or partial treatment of the lesion plus a 5-mm margin extending to the prostate capsule. Ablation targeted all suspicious areas on imaging or biopsy-confirmed cancer. Extra sonication sweeps were applied to MRI-visible tumors, with additional sweeps if undertreatment was suspected on MRI thermometry. Partial ablation was used for unilateral, well-confined lesions, while WG ablation was chosen for diffuse or extensive disease.

### 2.4 Follow-up, assessment, and endpoints

Follow-up visits were scheduled at 1–2 wk; then at 3, 6, 9, and 12 mo; and every 6 mo thereafter up to 5 yr. Catheter removal was attempted at the first visit. Multiparametric MRI was performed at 3 mo. At each visit, adverse events (AEs) were recorded using the Clavien-Dindo classification. Additional assessments included PSA levels, uroflowmetry, and quality-of-life (QoL) questionnaires (Expanded Prostate Cancer Index Composite [EPIC]-26, International Prostate Symptom Score [IPSS], IPSS QoL, and International Index of Erectile Function [IIEF]-5).

At 12 mo, patients underwent ^18^F-PSMA-1007 PET-CT and mpMRI, followed by outpatient cystoscopy and transrectal ultrasound-guided biopsy. The biopsy protocol included two to four in-field biopsies, with additional two to four biopsies per lesion identified as a Likert/PI-RR score of ≥3 or focal PSMA uptake activity higher than the surrounding prostate tissue. Cognitive guidance using visual registration from MRI and PSMA PET was employed for targeting suspicious areas. Longer-term assessments included clinical evaluations and PSA testing. PSMA PET-CT was performed if BCR was detected or metastatic progression was suspected.

The primary safety endpoint was the occurrence of severe GU and gastrointestinal (GI) toxicities, defined as any Clavien-Dindo ≥3 event or toxicity requiring hospitalization. The primary efficacy endpoint was the rate of histopathologically confirmed disease in the targeted area and within the entire prostate at 12 mo.

The secondary endpoints included leak- and pad-free continence at 12 mo, evaluated using EPIC-26 questions 1 (leak free: score ≥3) and 3 (pad free: score 0). Other secondary endpoints included overall survival (OS), metastasis-free survival (MFS), failure-free survival (FFS, defined as freedom from local salvage or systematic treatment, or metastases), and Phoenix BCR-free survival.

### 2.5 Sample size estimation

The inclusion of 40 patients balanced data collection with ethical and logistical consideration. Given the limited published data on sTULSA and the high-risk nature of salvage treatments for radiorecurrent PCa, a cautious approach with stringent inclusion criteria was necessary. The phase 1 study demonstrated feasibility and safety, supporting expanded enrollment. This sample size aims to provide meaningful data while addressing recruitment challenges through multicenter collaboration and an extended recruitment period, aligning with the exploratory nature of phase 2 studies.

### 2.6 Statistical analysis

All statistical analyses were performed using R (version 4.2.2; RStudio, PBC, Boston, MA, USA). Descriptive statistics summarized patient, treatment, and follow-up data, and continuous variables were visualized with box plots. Categorical variables were presented as frequencies and percentages, and continuous variables as medians with interquartile ranges (IQRs) or means with standard deviations, depending on distribution. Normality of continuous variables was assessed using the Shapiro-Wilk test and corroborated by visual inspection. Association between the ablation pattern (WG vs partial ablation) and the number of AEs, analyzed as both continuous and categorical variables, was assessed using the Wilcoxon rank-sum test and Pearson's chi-square test.

Nonparametric methods were used for non-normally distributed continuous variables. Associations over time were analyzed using repeated measures analysis of variance for normally distributed variables and Friedman test for non-normal distributions. Significant differences were examined further using paired *t* tests or Wilcoxon signed-rank tests, as appropriate.

Survival analyses were conducted using the Kaplan-Meier method, with 2- and 5-yr survival probabilities reported with 95% confidence intervals (CIs). The log-rank test was used to compare survival outcomes and severe GU toxicity between ablation patterns (WG vs partial ablation). Missing data were not imputed; analyses were performed on observed cases only. A two-sided *p* value of <0.05 was considered statistically significant.

## 3 Results

### 3.1 Patient characteristics

Between April 2018 and March 2023, 39 patients underwent sTULSA, with a median follow-up of 40 mo (IQR 24–55; Supplementary Fig. 1). At enrollment, the median age was 73 yr (IQR 69–77), median PSA 3.3 ng/ml (IQR 2–6.2), and median prostate volume on MRI 19 cm^3^ (IQR 16–24). Of the patients, 41% (*n* = 16) were receiving hormonal therapy and 10% (*n* = 4) had nonmetastatic castration-resistant PCa (Table 1, and Supplementary Tables 1 and 2). A total of 56 biopsy-proven tumors were detected across 39 patients, 52 of which were MRI visible (PI-RR ≥3) [22]. Baseline cystoscopy revealed incidental bulbar urethral stenosis/stricture in 18 patients (46%), with three requiring urethrotomy before sTULSA.

**Table 1:** Summary statistics of patient and radiorecurrent disease characteristics at enrollment

Characteristic	*N* = 39 a
Age	73.0 (69.0, 76.5)
Charlson comorbidity index	6 (5, 6)
PSA (ng/ml)	3.3 (2.0, 6.2)
EAU BCR high-risk group	8 (21)
Hormonal therapy at enrollment	16 (41)
Duration of hormonal therapy (mo)	38 (10, 87)
Patients with multifocal disease	13 (33)
Global MRI T stage	
T2	36 (95)
T3	2 (5)
PI-RR score	
3	3 (6)
4	15 (29)
5	34 (65)
Tumor diameter on MRI (mm)	13 (9, 18)
SUVmax on PSMA PET	15 (8, 23)
Total length of biopsy material (mm)	70 (46, 81)
Total length of cancer in biopsy (mm)	15 (7, 26)
Global ISUP GG	
2	5 (9)
3	19 (34)
4	14 (25)
5	18 (32)

BCR = biochemical recurrence; EAU = European Association of Urology; ISUP GG = International Society of Urological Pathology grade group; IQR = interquartile range; MRI = magnetic resonance imaging; PI-RR = Prostate Imaging Recurrence Reporting; PSA = prostate-specific antigen; PSMA PET = prostate-specific membrane antigen positron emission tomography; SUVmax = maximum standardized uptake value.

At enrollment, six patients were receiving medical castration therapy, five were on antiandrogens, and five were undergoing total androgen blockade (TAB). The median duration of medical castration therapy or TAB was 11 mo (IQR 6–79), while the median duration of antiandrogen therapy was 72 mo (IQR 37–137).

a Median (IQR); *n* (%).

The primary disease and RT characteristics are provided in Supplementary Tables 3 and 4. Of the patients, 97% had received EBRT, 64% with fiducial marker guidance, and 64% had adjuvant hormonal therapy. The median time from RT to sTULSA was 136 mo (IQR 106–155). Two patients had prior salvage HDRb, and one had salvage lymph node dissection. Prior urological interventions were common (31%), with notable procedures including transurethral resection of the prostate (*n* = 8), urethral dilation/optic urethrotomy (*n* = 4), transurethral cystolithotomy (*n* = 1), attempted but aborted RP (*n* = 1), and transurethral resection of the bladder (*n* = 1).

### 3.2 Study intervention

Salvage TULSA was feasible in all patients, with a median ablation time of 48 min (IQR 27–54; Supplementary Table 5). WG ablation was performed in 25 patients (64%). Most patients received a suprapubic catheter (SPC; *n* = 30), with the rest receiving a transurethral catheter. Overall, the median duration of catheterization was 18 d (IQR 13–28). One patient was discharged on the 2nd postoperative day, while the rest were discharged on the 1st day.

### 3.3 Safety

Table 2 summarizes the AEs associated with sTULSA. A total of 41 AEs were reported in 22 patients (56%). The most common AEs were urinary retention (UR) and urinary tract infections (UTIs; *n* = 29), followed by puboprostatic fistula (*n* = 3, median onset 23 mo, range 4–30), pelvic pain (*n* = 3), epididymitis (*n* = 3), bulbar urethral stricture (*n* = 2), and dystrophic calcification (*n* = 1).

**Table 2:** Adverse events associated with the salvage MRI-guided transurethral ultrasound ablation

Characteristic	*N* = 39 a
Overall no. of adverse events	41
Overall no. of adverse events <90 d	21
1	5 (24)
2	15 (71)
3a	1 (5)
Type of adverse events <90 d	
Urinary retention and urinary tract infection	10 (48)
Urinary tract infection	6 (29)
Pelvic pain	3 (14)
Urinary retention	2 (10)
Overall no. of adverse events >90 d	20
2	17 (85)
3b	3 (15)
Type of adverse events >90 d	
Urinary tract infection	6 (30)
Urinary retention and urinary tract infection	4 (20)
Epididymitis	3 (15)
Puboprostatic fistula	3 (15)
Bulbar urethral stricture	2 (10)
Urinary retention	1 (5)
Dystrophic calcification	1 (5)

IQR = interquartile range; MRI = magnetic resonance imaging.

Safety data were collected prospectively through chart reviews and face-to-face interviews conducted by clinical investigators. The frequency and severity of adverse events were recorded at each follow-up visit using the Clavien-Dindo classification for surgical complications.

No intraoperative complications occurred, and the early postoperative recovery following salvage MRI-guided transurethral ultrasound ablation was uneventful. Only genitourinary (GU) toxicity was observed, with no reports of gastrointestinal toxicity. Importantly, nine of the 11 patients who experienced severe GU toxicity had at least one of the following findings: a history of local salvage therapy (*n* = 1), significant transurethral interventions (*n* = 3), or a bulbar urethral stricture (*n* = 6) noted on baseline cystoscopy. Additionally, ten of these 11 patients had received whole-gland ablation.

a Median (IQR); *n* (%).

Five grade 3 AEs occurred in four patients, all receiving WG ablation: one UR treated with SPC and stents; one puboprostatic fistula requiring cystectomy with Bricker diversion; and two exacerbated bulbar urethral strictures, one of which also required cystectomy with Bricker diversion due to extensive dystrophic calcification in the bladder and prostate cavity. Both patients who underwent cystectomy recovered fully after initial conservative treatments, including SPC and antibiotics, were unsuccessful. Two of the puboprostatic fistulas were treated conservatively with prolonged SPC and antibiotics. Severe GU toxicity occurred in 28% (11/39) of patients, with ten having received WG ablation (Supplementary Table 6). There was a statistically significant difference (*p* = 0.012) between WG and partial ablation in severe GU toxicity, although no significant difference was observed in the total number of AEs (Supplementary Tables 7 and 8).

### 3.4 Functional, QoL, and uroflowmetry outcomes

Table 3 details the functional, QoL, and uroflowmetry outcomes over the 12-mo follow-up. The IPSS and EPIC-26 irritative/obstructive domains worsened at 3 mo, but improved by 12 mo, although neither returned to baseline (IPSS from 6 to 10, EPIC-26 irritative/obstructive from 75 to 71). The IPSS QoL worsened from 2 to 3. The EPIC-26 urinary incontinence domain dropped from 88 to 61, with leak- and pad-free continence maintained in 53% (16/30) and 47% (14/30) of patients, respectively. The bowel and hormonal domains showed slight improvement. Erectile function remained low from baseline throughout the follow-up. The maximum urinary flow rate declined from 13.6 to 10.5 ml/s, while postvoid residual remained stable (Supplementary Table 9 and Supplementary Fig. 2–4).

**Table 3:** Functional status, quality-of-life, uroflowmetry, and PSA outcomes during 12-mo follow-up

Characteristic	Baseline a (*n* = 39)	3 mo a (*n* = 39)	6 mo a (*n* = 39)	9 mo a (*n* = 39)	12 mo a (*n* = 39)	*p* value overall time b	*p* value t0 to t12 c
IPSS urinary symptom score	6 (3, 12)	14 (6, 23)	12 (7, 20)	14 (7, 17)	10 (4, 18)	0.001	0.326
IPSS quality of life	2 (1, 3)	3 (2, 4)	3 (2, 4)	2 (2, 3)	3 (2, 3)	0.001	0.205
IIEF-5 erectile function	0 (0, 2)	0 (0, 0)	0 (0, 0)	0 (0, 2)	0 (0, 2)	0.011	1.00
EPIC-26 urinary incontinence domain	88 (78, 94)	56 (38, 76)	69 (40, 81)	56 (38, 79)	61 (31, 86)	0.020	0.422
EPIC-26 irritative/obstructive domain	75 (75, 88)	69 (46, 75)	75 (48, 81)	69 (52, 77)	71 (46, 76)	0.226	
EPIC-26 sexual domain	17 (9, 21)	15 (4, 17)	15 (8, 17)	17 (8, 20)	17 (8, 17)	0.138	
EPIC-26 bowel domain	92 (86, 100)	96 (88, 100)	92 (81, 100)	92 (85, 100)	96 (88, 100)	0.224	
EPIC-26 hormonal domain	95 (85, 100)	95 (85, 100)	93 (84, 100)	95 (83, 100)	100 (93, 100)	0.043	0.208
Voided volume	256 (166, 409)	152 (91, 233)	154 (92, 215)	NA	182 (108, 254)	<0.001	0.086
Qmax	13.6 (11.1, 17.3)	7.7 (5.5, 11.4)	8.2 (6.5, 11.3)	NA	10.5 (6.3, 11.6)	<0.001	0.107
Qave	5.80 (4.20, 8.50)	3.80 (2.40, 5.10)	4.10 (2.80, 5.90)	NA	4.70 (3.80, 6.10)	0.001	0.584
PVR	32 (5, 96)	38 (16, 73)	32 (11, 91)	NA	23 (8, 83)	0.393	
PSA	3.30 (2.00, 6.15)	0.05 (0.01, 0.31)	0.10 (0.02, 0.19)	0.12 (0.05, 0.22)	0.17 (0.05, 0.29)	<0.001	<0.001

ANOVA = analysis of variance; EPIC-26 = Expanded Prostate Cancer Index-26; IIEF = International Index of Erectile Function; IPSS = International Prostate Symptom Score; NA = not available; PSA = prostate-specific antigen; PVR = postvoid residual volume; Qave = average urinary flow rate; Qmax = maximum urinary flow rate.

a Median (IQR).

b Repeated measures ANOVA; Friedman test.

c Paired *t* test; Wilcoxon signed rank test.

### 3.5 Oncological outcomes—histopathology, imaging, and PSA

Biopsy results at 12 mo are summarized in Table 4 and detailed in Supplementary Table 10. Of 203 biopsies performed across 37 patients, seven were positive in the targeted area and 17 were positive across the entire prostate. Most patients showed no evidence of cancer, with 89% being cancer free in the targeted area and 78% across the prostate. MRI and PSMA PET-CT results were negative for cancer in 92% of patients within the prostate and 79% overall (Table 5).

**Table 4:** Oncological efficacy at 12 mo—biopsy and prostate imaging outcomes

Outcome	Targeted area only	Whole prostate gland
Biopsy characteristics (*n* = 37 a)		
No. of biopsies taken per patient	4 (4, 6)	5 (4, 6)
Overall no. of biopsies taken	180	203
Overall no. of positive biopsies	7	17
Overall length of biopsy material (mm)	2045	2339
Overall length of cancer tissue (mm)	12.8	27
Biopsy histopathology (*n* = 37 a)		
No evidence of cancer	33 (89)	29 (78)
Grade group 1	0	1 (3)
Grade group 2	2 (5)	3 (8)
Grade group 3	1 (3)	1 (3)
Grade group 4	0	2 (5)
Grade group 5	1 (3)	1 (3)
Prostate only imaging outcome (*n* = 38 a)		
Both imaging modalities negative for cancer	36 (95)	35 (92)
MRI negative for cancer	37 (97)	36 (95)
PSMA PET-CT negative for cancer	36 (95)	36 (95)

CT = computed tomography; IQR = interquartile range; MRI = magnetic resonance imaging; PET = PET = prostate-specific membrane antigen positron emission tomography; PSMA = prostate-specific membrane antigen.

Biopsy protocol included two to four in-field biopsies with additional out-of-field biopsies taken only if imaging revealed suspicious findings. The median biopsy density of 10 mm/cm^3^ (IQR 5–24), calculated as the total biopsy length divided by the prostate volume on MRI, reflects the thoroughness of sampling. Supplementary Table 10 provides detailed additional information on oncological efficacy.

a Median (IQR); *n* (%).

**Table 5:** Oncological efficacy at 12 mo—overall and extraprostatic imaging status

Outcome	Overall	SV status	N status	M status
Imaging outcome (*n* = 38 a)				
Both imaging modalities negative for cancer	30 (79)	32 (84)	36 (95)	37 (97)
Pelvic MRI negative for cancer	34 (87)	35 (92)	38 (100)	38 (100)
wbPSMA PET-CT negative for cancer	31 (82)	32 (84)	36 (95)	37 (97)

IQR = interquartile range; MRI = magnetic resonance imaging; M status = distant metastasis status; N status = nodal status; PSMA PET-CT = prostate-specific membrane antigen positron emission tomography computed tomography; SV = seminal vesicles; wb = whole body.

a *n* (%).

PSA levels decreased by 95% from baseline to 12 mo (*p* < 0.001), with 15 patients reaching undetectable levels (Table 3 and Supplementary Fig. 5). This occurred despite 94% of patients discontinuing hormonal therapy after sTULSA. Among those who stopped ADT, 80% reached serum testosterone levels above the castrate threshold (>1.7 nmol/l), with a median of 11 nmol/l (IQR 5–19). MRI showed a median prostate volume reduction of 65% (IQR 50–96) overall and 94% (IQR 63–97) for WG ablation patients (Supplementary Table 11).

### 3.6 Survival outcomes

**Fig. 1:**
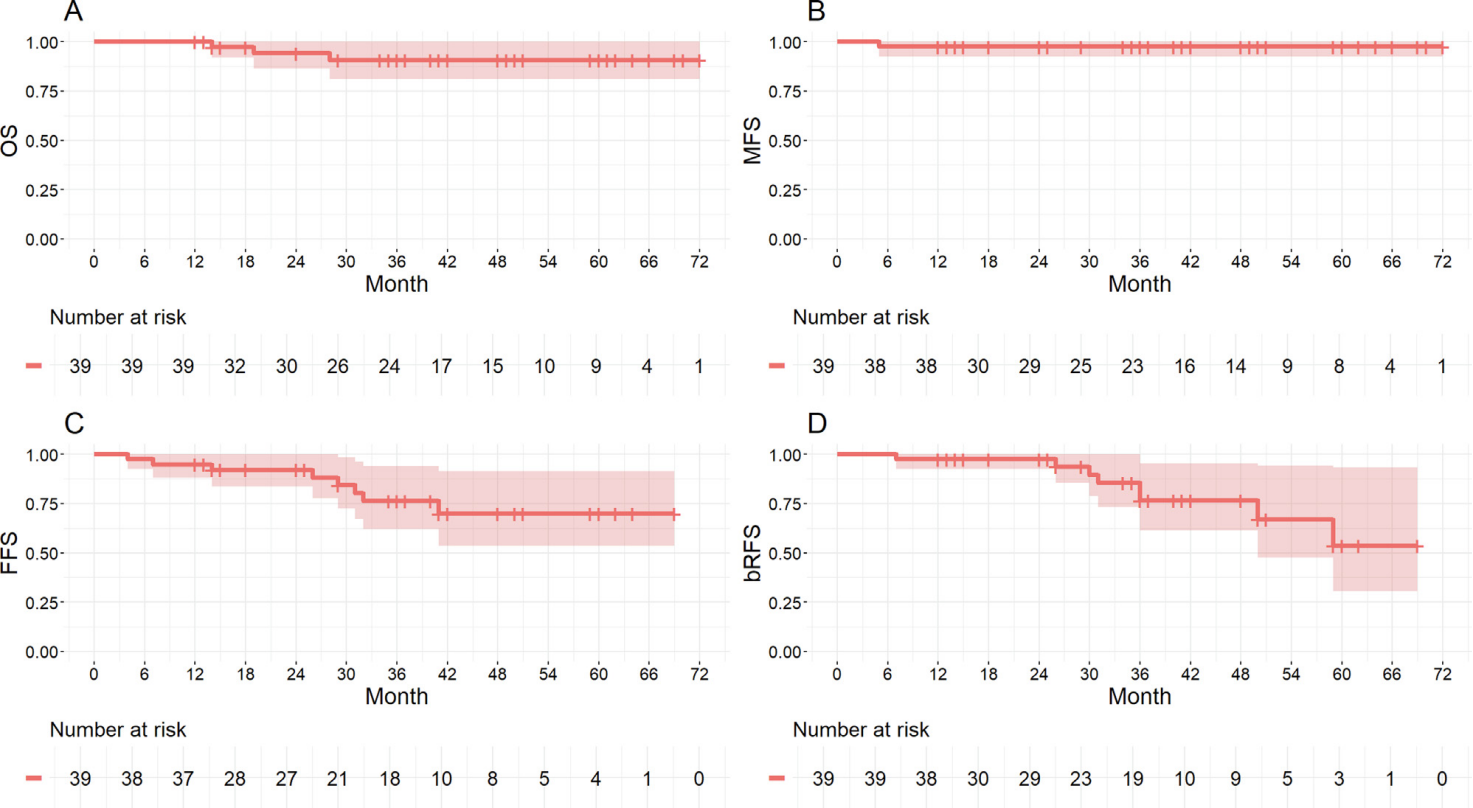
Survival outcomes: bRFS is freedom from prostate-specific antigen (PSA) rise above the nadir + 2, while FFS is freedom from local salvage, systemic treatment, or metastases. By the last follow-up (median 40 mo, IQR 24–55), 24 patients (62%) had negative biopsy and follow-up imaging, with no events in bRFS, FFS, or MFS. Including deaths unrelated to prostate cancer (PCa), the number of patients was 21 (54%). Six patients were on watchful waiting: five due to minimal PCa on biopsy with no BCR or extraprostatic disease on imaging, and one with slowly rising PSA just above BCR level, but no signs of disease on biopsy or imaging. BCR = biochemical recurrence; bRFS = biochemical recurrence–free survival; FFS = failure-free survival; IQR = interquartile range; MFS = metastasis-free survival; OS = overall survival.

Figure 1 displays the survival outcomes. During follow-up, three patients died without evidence of PCa: one from lung cancer, one from cardiovascular complications following endovascular aortic aneurysm repair, and one from heart failure due to ischemic heart disease. The 2- and 5-yr survival probabilities (95% CI) were as follows: OS, 0.94 (0.86–1.00) and 0.91 (0.81–1.00); MFS, 0.97 (0.93–1.00) and 0.97 (0.93–1.00); FFS, 0.92 (0.84–1.00) and 0.70 (0.54–0.91); and BCR–free survival, 0.97 (0.93–1.00) and 0.54 (0.31–0.93), respectively. There was no statistically significant difference between WG and partial ablation in any of the survival outcomes (Supplementary Table 12).

## 4 Discussion

This phase 2 study demonstrates the efficacy of sTULSA for radiorecurrent PCa, showing significant local control. However, given the notable incidence of severe GU toxicity, sTULSA should be approached with caution outside of clinical trials.

Up to 50% of patients develop BCR after RT, often leading to anxiety and potential progression to significant disease [1], [25], [26]. Balancing the need to delay metastasis or achieve a cure while avoiding overtreatment is crucial. Despite the prevalence of local recurrence, few patients undergo salvage therapy due to high toxicity, limited experience, and availability, often resulting in reliance on noncurative ADT with harmful side effects [27], [28], [29], [30]. In our study, 41% of patients had previously received only ADT, despite having prostate-confined recurrence.

The oncological outcomes are encouraging, with a 95% reduction in PSA levels at 12 mo, 89% of patients showing no cancer on biopsy in the targeted area, and 78% being cancer free on biopsy across the entire prostate. Imaging outcomes align with these findings, with MRI and PSMA PET-CT results being negative for cancer in 92% of cases within the prostate. The 5-yr MFS and FFS probabilities of 97% and 70%, respectively, demonstrate the treatment’s efficacy. However, these survival results may reflect the extended time from RT to sTULSA (median >11 yr), suggesting more indolent disease. In addition, the Phoenix criteria used for BCR may miss cases of micrometastasis, even with the use of PSMA PET imaging, potentially underestimating disease progression in some patients. Although some deaths occurred during follow-up, none were due to PCa, underscoring the challenge of accurately assessing life expectancy in this population.

Compared with other salvage therapies, our findings are promising. Valle et al’s [6] meta-analysis reported 5-yr recurrence-free survival (RFS) rates of 50% for cryotherapy and 60% for HDRb and SBRT, with no significant differences from salvage RP. However, limitations of the included studies, such as small sample size, retrospective design, variability in the definitions of BCR and radiorecurrent disease, and grouping of BCR-free survival and MFS under RFS, complicate direct comparisons. Despite these issues, our 5-yr FFS probability of 70% suggests that sTULSA offers comparable oncological control.

Toxicity remains a concern, with 56% of patients experiencing AEs, mostly of low to moderate severity, including UR and UTIs, likely due to prolonged prostate edema and necrotic tissue resolution, which may be exacerbated by the unique transurethral ablation technique used in TULSA [31]. Managing catheterization is challenging, as both early removal and prolonged use increase the risk of UR and UTIs. The median catheterization duration was 18 d, although the optimal timing and catheter type remain unclear. Urinary incontinence worsened in half of the patients, reflecting the complexity of managing radiorecurrent PCa. Severe GU toxicity occurred in 28% of patients, with no GI toxicity reported. The three cases of puboprostatic fistulas, all occurring in patients who underwent WG ablation with two full prostate-covering sonication sweeps, suggest a link to aggressive anterior treatment. We hypothesize that the anterior prostate is more susceptible to thermal injury in previously radiated prostates due to the lack of natural cooling, as radiation-induced obliteration of the anterior venous plexus may diminish heat dissipation. In contrast, the posterior region benefits from the protection of an endorectal cooling device, which helps shield the rectum and surrounding perirectal tissue from excessive heat. This may explain the increased risk of fistula formation in radiorecurrent cases, as no puboprostatic fistulas have been reported in the treatment of nonradiated prostates. A further analysis is ongoing to determine the relationship between treatment intensity and fistula formation. In the meantime, we recommend avoiding additional sonication anteriorly if the initial sonication is deemed sufficient based on MRI thermometry, to minimize the risk of fistula formation.

Our study’s GU toxicity rates exceeded those reported for HDRb and SBRT, which Valle et al [6] identified as the most tolerable salvage methods. These higher toxicity rates are likely attributable to the sTULSA technique. Other contributing factors might include the impact of our old patient population, prior transurethral procedures, previous salvage treatments, and baseline urethral strictures. Additionally, heterogeneity in the definitions and reporting of AEs across studies further complicates direct comparisons between salvage treatments.

The significant GU toxicity underscores the need for refining patient selection, particularly in those with prior salvage therapies, transurethral operations, or urethral strictures [32], [33]. Optimizing treatment parameters, including ablation extent, margins, thermal dosing, and catheterization protocols, is critical. In our study, an extra sonication sweep was applied to the tumor, although a single successful sonication sweep may suffice, potentially reducing toxicity. Long-term follow-up is essential to assess late-onset effects and the durability of oncological outcomes**.** This study also highlights TULSA’s versatility as a salvage therapy, effective for both partial and WG ablation, with a transurethral approach suitable for treating hard-to-reach tumors. However, the relatively small median prostate size of 19 cm^3^ in this cohort may have contributed to the favorable outcomes.

While the study has limitations, including its single-arm design, moderate sample size, single-center setting, and limited follow-up, it is strengthened by its prospective design. The use of PSMA PET for precise patient selection and outcome assessment, along with comprehensive medical histories and follow-up, enhances the study’s rigor. Overall, these results improve our understanding of sTULSA, and aid in patient counseling. Further studies are required to refine the modality and improve its therapeutic ratio before it can be considered a standard local salvage treatment option.

## 5 Conclusions

Salvage TULSA demonstrates excellent effectiveness for radiorecurrent PCa, although significant GU toxicity remains a concern, requiring caution when used outside clinical trials. Optimizing patient selection, treatment parameters, and postablation management is essential for enhancing safety. Further studies should focus on refining these factors and extending follow-up to improve the tolerability and long-term effectiveness. These efforts will be crucial in maximizing sTULSA’s therapeutic potential while minimizing adverse effects, ultimately providing a safer and more effective salvage option for radiorecurrent PCa patients.

  ***Author contributions*:** Mikael Anttinen had full access to all the data in the study and takes responsibility for the integrity of the data and the accuracy of the data analysis.

  *Study concept and design*: Anttinen, Blanco Sequeiros, Boström.

*Acquisition of data*: Anttinen, Mäkelä, Malaspina, Pärssinen, Pertti Nurminen, Teija Sainio, Mikael Högerman, Pekka Taimen.

*Analysis and interpretation of data*: Anttinen, Högerman, Boström.

*Drafting of the manuscript*: Anttinen, Högerman.

*Critical revision of the manuscript for important intellectual content*: Mäkelä, Nurminen, Högerman, Taimen, Blanco Sequeiros, Boström.

*Statistical analysis*: Anttinen, Högerman.

*Obtaining funding*: Anttinen, Boström, Blanco.

*Administrative, technical, or material support*: Boström, Taimen, Mäkelä, Pärssinen, Sainio, Malaspina, Blanco Sequeiros.

*Supervision*: Taimen, Boström, Blanco Sequeiros.

*Other*: None.

  ***Financial disclosures:*** Mikael Anttinen certifies that all conflicts of interest, including specific financial interests and relationships and affiliations relevant to the subject matter or materials discussed in the manuscript (eg, employment/affiliation, grants or funding, consultancies, honoraria, stock ownership or options, expert testimony, royalties, or patents filed, received, or pending), are the following: Dr. Mikael Anttinen reports grants and honorariums from Profound Medical Inc, TYKS Foundation, Ida Montini Foundation, Emil Aaltonen Foundation, Finnish Urological Research Foundation, and Finnish Urological Association, outside the submitted work. Dr. Pietari Mäkelä reports grants from TYKS Foundation, the Finnish Radiological Society, the Finnish Medical Society Duodecim, and the Cancer Foundation Finland, all outside the submitted work. Dr. Pekka Taimen reports grants from the Cancer Foundation Finland; personal fees from Roche, AstraZeneca, and MSD; and nonfinancial support from MSD, all outside the submitted work. Dr. Peter J. Boström reports grants from the Cancer Foundation Finland, and personal fees from Profound Medical Inc and Janssen-Cilag Company, outside the submitted work. The other authors declare that there is no conflict of interest regarding the publication of this article.

  ***Funding/Support and role of the sponsor*:** None.

  ***Acknowledgments*:** We thank all the patients and referring physicians whose participation made this study project possible. We thank the entire staff team in the Departments of Medical Physics, Urology, Pathology, Diagnostic Radiology, Clinical Physiology, and Nuclear Medicine and the Turku PET Centre at the Turku University Hospital. We also want to thank the staff team of the urological outpatient clinic at Turku University Hospital for its contribution to the project. Without their help and support, timely completion of this project would not have been possible.

  ***Data sharing statement*:** All the data collected for the study, including the deidentified individual participant data, and the study protocol and informed consent forms (in Finnish) will be available for anyone who wishes to access the data for a period commencing with the publication and ending 5 yr later. Proposals for access to the data should be directed to mikael.hogerman@varha.fi to gain access to the data. Requestors will need to sign a data access agreement.
