# Supplementary material for: Salvage Magnetic Resonance Imaging–guided Transurethral Ultrasound Ablation for Localized Radiorecurrent Prostate Cancer
Source: Eur Urol Open Sci. 2024 Dec 5;71:69–77. doi: 10.1016/j.euros.2024.11.001 (PMC11656090; doi:10.1016/j.euros.2024.11.001)
Supplement: Supplementary Data 1 [file mmc1.docx]

**Supplementary material**

**Table of contents**

[**Detailed description of the MRI-guided transurethral ultrasound ablation (TULSA) technique: Study intervention and treatment strategy** 3](#_Toc184212692)

[**Supplementary Figure 1. Study flow chart** 4](#_Toc184212693)

[**Supplementary Figure 2. Box plot of uroflowmetry outcomes during 12 months follow-up** 5](#_Toc184212694)

[**Supplementary Figure 3. Box plots of IPSS, IPSS QoL and IIEF** 6](#_Toc184212695)

[**Supplementary Table 1. Detailed radiorecurrent disease characteristics of individual patients before salvage MRI-guided transurethral ultrasound ablation** 9](#_Toc184212696)

[**Supplementary Table 2. Detailed patient characteristics of individual patients at salvage MRI-guided transurethral ultrasound ablation** 10](#_Toc184212697)

[**Supplementary Table 3. Summary statistics of primary disease characteristics and disease history before salvage MRI-guided transurethral ultrasound ablation** 11](#_Toc184212698)

[**Supplementary Table 4. Detailed disease history of individual patients before salvage MRI-guided transurethral ultrasound ablation** 12](#_Toc184212699)

[**Supplementary Table 5. Treatment parameters and perioperative outcomes related to salvage MRI-guided transurethral ultrasound ablation** 13](#_Toc184212700)

[**Supplementary Table 6. Adverse events after salvage MRI-guided transurethral ultrasound ablation: Bolded events attributable to the study intervention** 14](#_Toc184212701)

[**Supplementary Table 7. Comparison of severe genitourinary toxicity based on ablation pattern (whole-gland vs. partial gland ablation)** 15](#_Toc184212702)

[**Supplementary Table 8. Association between the ablation pattern (whole-gland vs. partial gland ablation) and the number of adverse events** 16](#_Toc184212703)

[**Supplementary Table 9. Uroflowmetry outcomes during 12 months follow-up** 17](#_Toc184212704)

[**Supplementary Table 10. Oncological outcomes at 12 months after salvage MRI-guided transurethral ultrasound ablation.** 18](#_Toc184212705)

[**Supplementary Table 11. The evolution of prostate volume and non-perfused volume on mpMRI after salvage MRI-guided transurethral ultrasound ablation** 19](#_Toc184212706)

[**Supplementary Table 12. Comparison of survival outcomes based on ablation pattern (whole-gland vs. partial gland ablation)** 20](#_Toc184212707)

**Detailed description of the MRI-guided transurethral ultrasound ablation (TULSA) technique: Study intervention and treatment strategy**

*TULSA-PRO® System (Profound Medical Inc., Mississauga, Canada)*

The TULSA system features a semirigid 22 French transurethral ultrasound applicator (UA), equipped with a coude-tip and a central channel for guidewire insertion. The UA has a 50 mm active length comprising 10 single-element transducers (each 5 mm long and 4 mm wide), emitting high-intensity directional ultrasound energy from within the prostatic urethra to adjacent prostatic tissue. Each transducer operates independently at either 4 or 13 MHz with acoustic power ranging from 0 to 4 W.

*Cooling and Positioning*

The system uses a fluid circuit to cool the urethra and rectum via the UA and an endorectal cooling device (ECD), protecting up to 2 mm of urethral tissue and the rectum wall. The UA attaches to a magnetic resonance (MR)-compatible positioning system (PS) fixed to an MR-patient table, enabling robotic control and rotational motion to target specific regions during treatment.

*User Interface and Real-Time Monitoring*

The TULSA system includes a user interface for registering the UA on magnetic resonance imaging (MRI), contouring the target prostate volume, and monitoring real-time thermal therapy delivery. A proprietary temperature feedback control algorithm adjusts the ultrasound output, rotation rate, and frequencies based on updated thermometry images received every 6 seconds.

*Patient Preparation*

Patients fasted for 8 hours and underwent bowel preparation with oral Bisacodyl the day before and a Bisacodyl enema on the morning of the procedure. Single-dose prophylactic levofloxacin 500 mg was administered intravenously. The procedure was performed in the MRI suite with the patient in a supine position under general anesthesia.

*Device Instrumentation*

On the MR-table, an ECD was inserted into the rectum. After preloading the urethra with 2% lidocaine gel, a 16 French Foley catheter was inserted into the bladder. If a suprapubic catheter (SPC) was used, the bladder was filled with sterile saline before SPC insertion. A guidewire was inserted through the transurethral catheter into the bladder, followed by UA insertion over the guidewire.

*Intraprocedural Treatment Planning and Execution*

Treatment planning covered all suspicious areas identified by imaging or biopsies, with a 5 mm MRI-based healthy tissue margin. Two sonication sweeps per patient were performed to the tumor under real-time temperature feedback control, aiming for 55ºC at the prostate boundary. The controller targeted 57ºC at a control boundary 2 mm inside the prostate edge, expecting a cytocidal thermal dose of 240 Cumulative Equivalent Minutes (CEM).

*Post-Treatment Imaging*

Post-treatment MRI included dynamic images with high temporal resolution (70 scans in 4 min 55 s, spatial resolution 1.08 x 1.08 x 3.0 mm) and static contrast-enhanced images with high spatial resolution (0.78 x 0.78 x 1 mm). These sequences provided detailed perfusion information.

*Post-Therapy Care and Catheterization*

If no SPC was inserted, a 16 French Foley catheter was used post-therapy. Patients were monitored in the recovery room, ensuring free urine flow and adequate hydration with a urine output of at least 2 ml/kg/h. All patients were observed in the urological ward for at least one night following the intervention. Upon discharge, patients were prescribed paracetamol and nonsteroidal anti-inflammatory analgesics for use as needed. Catheter removal was scheduled within 1-2 weeks, with the timing and choice of catheter type influenced by factors such as the extent of treatment, patient preference, and bladder emptying capability at baseline. Post-void residual (PVR) volume was estimated to confirm adequate bladder emptying before catheter removal. Patients with an SPC kept a residual diary for 3 days before removal to ensure reliable bladder emptying.

*Diagnostic Workup and Selection of Treatment Strategy*

Patients with biochemical recurrence and MRI-visible disease after radiotherapy were recruited from multiple centers across the country, where the initial diagnostic workup for recurrent prostate cancer (PCa) was often initiated. Available diagnostic information, including imaging and biopsy data from these centers, was carefully re-reviewed and utilized, with any necessary complementary tests conducted at our institution to confirm eligibility.

Within 3 months prior to salvage TULSA, pre-TULSA pelvic 3T multiparametric MRI and fluorine-18 prostate-specific membrane antigen-1007 positron emission tomography-computed tomography (18F-PSMA-1007 PET-CT), along with MRI-targeted biopsies, were performed to histopathologically confirm the presence of disease. If partial treatment was planned, an additional systematic biopsy was strongly recommended but not mandatory.

The method of MRI-targeted biopsy varied between referral centers, utilizing either ultrasound-MRI fusion or cognitive fusion techniques via the transrectal route. At our institution, both pre- and post-salvage TULSA, MRI-targeted biopsies were uniformly performed using cognitive registration from MRI and targeting with transrectal ultrasound through the transrectal route, in accordance with our standard of care. All post-TULSA biopsies were performed at our center.

This diagnostic information was integral to treatment planning. Patients received either whole-gland (WG) or partial treatment based on factors such as dominant disease location, lesion diffusivity, size, overall disease burden, and patient preference. Ablation targeted all suspicious areas identified on imaging or biopsy-confirmed cancer, including a 5 mm margin around the tumor up to the prostate capsule.

An extra sonication sweep (boost) was applied to MRI-visible tumors to enhance ablation efficacy. If undertreatment was suspected based on MRI-thermometry images, additional sonication sweeps were performed to ensure complete ablation. The ablation created angular arc-like patterns, which varied between segmental, hemiablation, and WG ablation, depending on the disease extent and location. Partial ablation was only performed when the lesion was unilateral, well-confined, and concordant on both biopsy and imaging.

If patients were receiving hormonal treatment at the time of enrollment, it was discontinued no later than the 3-month follow-up visit post-TULSA to accurately assess the prostate-specific antigen response and avoid potential confounding effects of hormonal suppression.

# **Supplementary Figure 1. Study flow chart**

58 patients assessed for eligibility

19 patients excluded

Not meeting inclusion criteria (n=19)

- Hip prothesis (n=1)
- LDR brachytherapy seeds (n=4)
- Extraprostatic disease (n=14)
  - SVI (n=7)
  - Metastasis (n=7)

39 patients enrolled and received sTULSA treatment

38 patients completed 12-month MRI and PSMA PET-CT imaging

37 patients completed 12-month MRI-targeted biopsy

One patient biopsy omitted due to puboprostatic fistula

**Study intervention**

**Enrollment**

LDR = low dose rate; MRI = Magnetic resonance imaging; PSMA PET-CT = Prostate-specific membrane antigen positron-emission tomography-computed tomography; sTULSA = salvage MRI-guided transurethral ultrasound ablation; SVI = seminal vesicle invasion

* Patient withdrew after three months post-sTULSA due to the stringent follow-up protocol, despite having undetectable PSA at 3 and 12 months.

One patient withdrew consent*

**Follow-up**

# **Supplementary Figure 2. Box plot of uroflowmetry outcomes during 12 months follow-up**

PVR = post-void residual; Q-ave = average urinary flow rate; Q-max = maximum urinary flow rate

**Supplementary Figure 3. Box plots of IPSS, IPSS QoL and IIEF**

IIEF = International Index of Erectile Function [IIEF]-5; IPSS = International Prostate Symptom Score; QoL = quality of life (Q8)

**Supplementary Figure 4. Box plots of EPIC-26 domains**

EPIC-26 = Expanded Prostate Cancer Index Composite-26

**Supplementary Figure 5. Box plot of PSA outcomes**

PSA = prostate-specific antigen

**Supplementary Table 1. Detailed radiorecurrent disease characteristics of individual patients before salvage MRI-guided transurethral ultrasound ablation**

| **Pt** | **ADT at enrolment** | **Duration of ADT (mo)** | **MRI T-stage** | **PSA (ng/ml)** | **Prostate volume (cm^3^)** | **Positive Bx/Bx taken** | **Total length (mm)** | | **ISUP GG^c^** | **PI-RR score** | **Tumor diameter on MRI (mm)** | **SUVmax** |
| --- | --- | --- | --- | --- | --- | --- | --- | --- | --- | --- | --- | --- |
|  |  |  |  |  |  |  | **Bx** | **Cancer** |  |  |  |  |
| **1** | Bicalutamide | 37 | 2a  2a | 1.9 | 18 | 4/6^a^  3/6^a^ | 30 | 86 | 3  3 | 4  4 | 13  15 | 7.2  11.3 |
| **2** | - | - | 2a | 5.5 | 37 | 3/8^b^ | 70 | 12 | 5 | 4 | 8 | 6.8 |
| **3** | Bicalutamide | 137 | 2c  2a | 7.5 | 14 | 6/6^a^  4/6^a^ | 96  75 | 45  27 | 3  3 | 5  4 | 21  11 | 48.1  48.1 |
| **4** | - | - | 2a | 3.3 | 18 | 4/6^b^ | 84 | 8 | 5 | 4 | 11 | 44.6 |
| **5** | - | - | 2a | 16 | 24 | 3/3^b^ | 32 | 22 | 3 | 5 | 21 | 23.3 |
| **6** | - | - | 2a | 11 | 21 | 5/6^b^ | 59 | 28 | 3 | 5 | 17 | 5.4 |
| **7** | - | - | 2a  2a | 4.7 | 33 | 3/4^b^  4/4^b^ | 70  50 | 21  25 | 4  2 | 5  5 | 16  9 | 17.7  8.1 |
| **8** | Degarelix + Bicalutamide | 8 | 2a | 0.37 | 24 | 1/3^b^ | 33 | 2 | 4 | 4 | 11 | 7.4 |
| **9** | - | - | 3b  2a | 13 | 21 | 5/5^b^  2/4^b^ | 64  37 | 26  7 | 5  5 | 5  5 | 24  10 | 10.7  10.7 |
| **10** | - | - | 2c | 9.5 | 20 | Refused Bx | | | | 5 | 18 | 49.6 |
| **11** | Bicalutamide | 19 | NLD | 0.079 | 16 | 1/12^a^ | 165 | 8 | 3 | NLD | NLD | NLD |
| **14** | - | - | 2c | 3.8 | 20 | 4/4^b^ | 71 | 20 | 5 | 4 | 23 | 8.1 |
| **15** | - | - | 2a  2a | 4.8 | 24 | 2/3^b^  1/5^b^ | 84 | 3  1 | 4  4 | 4  5 | 7  6 | 5.6  9.2 |
| **16** | Degarelix + Cyproterone acetate | 39 | 2b | 9.2 | 10 | 4/4^b^ | 45 | 25 | 3 | 5 | 20 | 51.7 |
| **17** | - | - | 2a | 2.9 | 13 | 1/6^b^ | 55 | 4 | 2 | 5 | 15 | 8.5 |
| **18** | Goserelin + Bicalutamide | 102 | 2b | 2.0 | 25 | 8/9^a+b^  6/6^a^ | 128  99 | 37  10 | 4  4 | 5 | 24 | 81.9 |
| **19** | Bicalutamide | 180 | 2a | 3.3 | 18 | 0/3^a^  1/2^a^  1/3^b^ | 80 | 10 | 4 | 4 | 19 | 7.1 |
| **20** | - | - | 2a | 13 | 20 | 3/5^b^ | 50 | 15 | 5 | 5 | 22 | 22.7 |
| **21** | - | - | 2a | 2.9 | 31 | 3/6^a^  3/6^a^  2/2^b^ | 75  79  24 | 14  8  6 | 4  4  4 | 4 | 13 | 6.1 |
| **22** | - | - | 2a | 3.4 | 13 | 4/5^a^  2/6^a^  2/2^b^ | 68  89  24 | 19  2  8 | 4  4  4 | 5 | 14 | 10.8  12.7 |
| **23** | - | - | 2a  2a | 1.6 | 27 | 1/6^a^  0/6^a^ | 137 | 4 | 3 | 3  3 | 8  6 | 5.2  5.8 |
| **24** | - | - | 2a | 6.6 | 23 | 1/6^a^  6/6^a^  3/4^b^ | 73  76  39 | 2  19  5 | 3  3  3 | 4 | 10 | 5.3 |
| **25** | - | - | 2a | 2.6 | 16 | 4/4^b^ | 46 | 18 | 4 | 5 | 8 | 17.3 |
| **26** | - | - | 2a | 3.0 | 43 | 3/3^b^ | 35 | 15 | 5 | 4 | 11 | 5.8 |
| **27** | Bicalutamide | 72 | 2a  2a | 7.5 | 16 | 0/4^a^  0/4^a^  5/6^b^ | 35  38  55 | 0  0  11 | 2 | 4  4 | 4  6 | 13.8  13.8 |
| **28** | - | - | 2b | 5.7 | 19 | 4/6^a^  0/6^a^ | 87  77 | 48  0 | 5 | 5 | 22 | 21.9 |
| **29** | Leuprorelin | 130 | 2c | 1.6 | 19 | 6/6^a^  6/6^a^ | 60  63 | 45  39 | 5  5 | 5 | 30 | 13.8  9.3 |
| **30** | - | - | 2a | 2.8 | 19 | 1/6^a^  4/6^a^ | 85  88 | 1  30 | 5  5 | 5 | 20 | 22.4 |
| **32** | Leuprorelin + Bicalutamide | 82 | 2c | 3.3 | 21 | 3/7^a+b^  3/5^a+b^ | 65  50 | 10  10 | 3  3 | 5 | 27 | 15.7 |
| **33** | Degarelix | 2 | 2a  2c  2a | 2.8 | 17 | 0/4^a^  3/3^b^  0/4^b^ | 51  46  13 | 0  22  0 | 2 | 5  5  5 | 18  17  8 | 26  26  26 |
| **34** | - | - | 2a | 2.9 | 14 | 0/6^a^  0/6^a^  2/6^b^ | 79  85  94 | 0  0  1 | 2 | 4 | 9 | 15.2 |
| **36** | - | - | 2a  2a  2a | 2.0 | 19 | 3/3^b^  2/3^b^ | 53  39 | 18  5 | 3  3 | 5  5  3 | 8  11  7 | 13.4  8.5  NLD |
| **37** | Leuprorelin + Bicalutamide | 75 | 2a | 0.59 | 16 | 6/6^a^  0/6^a^ | 171 | 35 | 5 | 5 | 22 | 22.5 |
| **38** | - | - | 2a  2a | 3.3 | 21 | 5/6^a^  1/6^a^ | 68  81 | 39  3 | 5  5 | 5  5 | 17  9 | 20.2  8.1 |
| **39** | - | - | 2c  2c | 8.1 | 19 | 1/6^b^  3/3^b^ | 45  45 | 2  9 | 3  3 | 5  5 | 9  18 | 21.3  21.3 |
| **40** | Leuprorelin | 2 | 2b | 4.7 | 15 | 3/5^a^  0/7^a^ | 74  90 | 11  0 | 5 | 5 | 15 | 34.8 |
| **41** | Leuprorelin | 10 | 3a | 0.084 | 14 | 7/8^a+b^  0/6^a^ | 120  75 | 36  0 | 5 | 5 | 10 | 8.2 |
| **42** | Triptorelin | 4 | 2a | 0.46 | 28 | 2/4^b^ | 40 | 16 | 3 | 5 | 11 | 9.3 |
| **43** | Goserelin | 11 | 2a  2a | 0.84 | 42 | 0/6^a^  0/6^a^  6/6^b^ | 70  70  70 | 0  0  30 | 5 | 5  5 | 18  8 | 22.5 |

ADT = androgen deprivation therapy; Bx = biopsy; ISUP GG = International Society of Urological Pathology grade group; MRI = magnetic resonance imaging; NLD = no lesion detected; PI-RR = Prostate Imaging Recurrence Reporting; PSA = prostate-specific antigen; Pt = patient number; SUVmax = maximum standardized uptake value; TD = tumor diameter; TPV = total prostate volume

Green rows indicate pts with multifocal disease based on imaging and biopsy.

^a^ Systematic biopsy (first row right lobe and second row left lobe when applicable)

^b^ MRI-targeted biopsy.

^c^ Pathological determination of ISUP GG for salvage pts is not standardized because of radiation-induced changes.

All but one patient (No. 10), who refused biopsy and had an 18 mm Prostate Imaging Recurrence Reporting (PI-RR) 5 tumor on MRI with maximum standardized uptake value (SUVmax) 50 on PSMA PET-CT, had biopsy-proven recurrence.

There was one patient (No. 11) with a previously positive MRI and biopsy-proven disease but a negative baseline MRI, likely due to bicalutamide medication initiated after the diagnosis.

**Supplementary Table 2. Detailed patient characteristics of individual patients at salvage MRI-guided transurethral ultrasound ablation**

| **Pt** | **Age (yr)** | **Body mass index** | **ECOG-PS** | **Creatinine (µmol/l)** | **Charlson comorbidity Index** | **Anticoagulation** | **M0-CRPCa** | **EAU risk for BCR^1^** |
| --- | --- | --- | --- | --- | --- | --- | --- | --- |
| **1** | 69 | 29 | 0 | 86 | 4 | No | No | Low |
| **2** | 69 | 29 | 0 | 60 | 4 | No | No | Low |
| **3** | 69 | 38 | 0 | 91 | 6 | No | No | Low |
| **4** | 69 | 29 | 0 | 85 | 4 | No | No | High |
| **5** | 80 | 20 | 0 | 87 | 6 | No | No | Low |
| **6** | 77 | 21 | 1 | 96 | 7 | Acetylsalicylic acid | No | Low |
| **7** | 70 | 26 | 0 | 83 | 6 | No | No | Low |
| **8** | 66 | 29 | 0 | 83 | 4 | No | No | Low |
| **9** | 67 | 31 | 1 | 87 | 4 | Acetylsalicylic acid | No | High |
| **10** | 81 | 26 | 0 | 84 | 6 | Acetylsalicylic acid | No | Low |
| **11** | 62 | 32 | 0 | 75 | 4 | Acetylsalicylic acid | No | Low |
| **14** | 72 | 24 | 0 | 85 | 5 | No | No | Low |
| **15** | 76 | 26 | 0 | 85 | 6 | No | No | Low |
| **16** | 74 | 25 | 0 | 101 | 5 | No | Yes | Low |
| **17** | 64 | 24 | 0 | 62 | 5 | No | No | Low |
| **18** | 76 | 29 | 0 | 93 | 6 | Acetylsalicylic acid and dipyridamol | Yes | High |
| **19** | 73 | 26 | 0 | 121 | 5 | No | No | Low |
| **20** | 71 | 23 | 0 | 82 | 5 | No | No | Low |
| **21** | 72 | 30 | 0 | 93 | 5 | No | No | Low |
| **22** | 73 | 25 | 0 | 101 | 6 | Apixaban | No | High |
| **23** | 77 | 24 | 0 | 95 | 7 | Acetylsalicylic acid | No | High |
| **24** | 73 | 22 | 0 | 63 | 6 | Acetylsalicylic acid | No | Low |
| **25** | 73 | 25 | 0 | 74 | 5 | No | No | Low |
| **26** | 74 | 23 | 0 | 84 | 6 | No | No | High |
| **27** | 67 | 26 | 0 | 83 | 4 | No | No | Low |
| **28** | 56 | 33 | 0 | 88 | 4 | No | No | Low |
| **29** | 71 | 27 | 0 | 115 | 5 | No | No | Low |
| **30** | 78 | 28 | 0 | 90 | 5 | No | No | Low |
| **32** | 75 | 32 | 0 | 180 | 7 | Acetylsalicylic acid | Yes | Low |
| **33** | 80 | 23 | 0 | 86 | 6 | No | No | Low |
| **34** | 82 | 28 | 0 | 84 | 7 | Acetylsalicylic acid | No | Low |
| **36** | 70 | 32 | 0 | 105 | 5 | Acetylsalicylic acid | No | Low |
| **37** | 66 | 34 | 0 | 76 | 4 | Acetylsalicylic acid | Yes | Low |
| **38** | 81 | 22 | 0 | 102 | 6 | No | No | High |
| **39** | 79 | 28 | 1 | 116 | 9 | Apixaban | No | Low |
| **40** | 81 | 29 | 0 | 159 | 6 | No | No | Low |
| **41** | 75 | 28 | 0 | 91 | 6 | No | No | High |
| **42** | 72 | 28 | 0 | 119 | 6 | No | No | Low |
| **43** | 72 | 31 | 0 | 64 | 6 | No | No | Low |

BCR = Biochemical recurrence; CRPCa = castration resistant prostate cancer; EAU = European Association of Urology; ECOG-PS = Eastern cooperative oncology group performance status; Pt = patient number.

^1^ Patients are stratified into EAU categories with low or high risk of BCR after primary radiotherapy on the basis of International Society of Urological Pathology biopsy grade and the interval to BCR. ISUP grade 4–5 and a time interval from definitive radiotherapy to BCR of < 18 mo are considered high-risk features.

# **Supplementary Table 3. Summary statistics of primary disease characteristics and disease history before salvage MRI-guided transurethral ultrasound ablation**

| **Characteristic** | **N = 39**^1^ |
| --- | --- |
| **cT-stage** |  |
| T1 | 17 (44%) |
| T2 | 11 (28%) |
| T3 | 10 (26%) |
| T4 | 1 (3%) |
| **ISUP GG** |  |
| 1 | 20 (51%) |
| 2 | 9 (23%) |
| 3 | 2 (5%) |
| 4 | 2 (5%) |
| 5 | 6 (15%) |
| **PSA (ng/ml)** | 13 (10, 22) |
| **Primary RT type** |  |
| cf EBRT | 35 (90%) |
| hf EBRT | 3 (8%) |
| HDRb | 1 (3%) |
| **RT technique** |  |
| 3D-CRT | 13 (33%) |
| IMRT | 24 (62%) |
| VMAT | 1 (3%) |
| HDRb | 1 (3%) |
| **Total dose (Gy)** |  |
| cf EBRT | 74 (72, 76) |
| hf EBRT | 60 (60, 62) |
| HDRb | 27 |
| **Fiducial marker guided EBRT** | 25 (64%) |
| **(Neo)adjuvant hormonal therapy** |  |
| Antiandrogen | 5 (13%) |
| LHRH-agonist | 18 (46%) |
| TAB | 2 (5%) |
| **Duration of adjuvant hormonal therapy (mo)** | 12 (6, 36) |
| **Continuous hormonal therapy until sTULSA** | 2 (5%) |
| **PSA nadir (ng/ml) after pTx** | 0.4 (0.1, 0.75) |
| **Time from pTx to BCR (mo)** | 93 (57, 132) |
| **Highest post-RT PSA (ng/ml)** | 5.0 (3.0, 8.5) |
| **Local salvage therapy after primary RT** |  |
| sHDRb | 2 (5%) |
| Robotic-assisted laparoscopic sLND | 1 (3%) |
| **Time from pTx to sTULSA (mo)** | 136 (106, 155) |
| ^1^Median (IQR); n (%)  cf = conventional fractionation; EBRT = external beam RT; HDRb = salvage high dose rate brachytherapy; hf = hypofractionation; IMRT = intensity-modulated RT; IQR = interquartile range; ISUP GG = International Society of Urological Pathology grade group; LHRH = luteinizing hormone-releasing hormone; PSA = prostate-specific antigen; Pt = patient number; pTx = primary treatment; RT = radiation therapy; sLND = salvage lymph node dissection; sTULSA = salvage magnetic resonance imaging-guided transurethral ultrasound ablation; TAB = total androgen blockage; 3D-CRT = three-dimensional conformal RT; VMAT = volumetric modulated arc therapy  Supplementary Table 4 provides detailed information on each patient's characteristics at initial diagnosis and their disease history prior to undergoing sTULSA. | |

**Supplementary Table 4. Detailed disease history of individual patients before salvage MRI-guided transurethral ultrasound ablation**

| **Pt** | **Characteristics at pTx** | | | **Year of diagnosis** | **RT parameters** | | | | | **Neo/adjuvant hormonal therapy** | | **PSA nadir (ng/ml)** | **Time from pTx to BCR (mo)^a^** | **Highest PSA post-radiation (ng/ml)** | **Time from pTx to sTULSA (mo)** |
| --- | --- | --- | --- | --- | --- | --- | --- | --- | --- | --- | --- | --- | --- | --- | --- |
|  | **Clinical T-stage** | **ISUP GG** | **PSA (ng/ml)** |  | **Radiation type** | **Technique** | **Total Dose (Gy)** | **Fiducial seeds** | |  |  |  |  |  |  |
|  |  |  |  |  |  |  |  | **n** | **Material** | **Duration (mo)** | **Type** |  |  |  |  |
| **1** | T3 | 1 | 13 | 2006 | cf EBRT | IMRT | 78 | 3 | Gold | 6 | LHRH-agonist | 0.6 | 72 | 15 | 132 |
| **2** | T2 | 1 | 9 | 2005 | cf EBRT | 3D-CRT | 72 | 0 | - | 12 | Bicalutamide | 0.46 | 132 | 6 | 149 |
| **3** | T3 | 1 | 21 | 2007 | cf EBRT | 3D-CRT | 72 | 0 | - | Continuous | Bicalutamide | <0.1 | 108 | 9 | 132 |
| **4** | T2 | 5 | 10 | 2009 | cf EBRT | 3D-CRT | 72 | 0 | - | 36 | LHRH-agonist | 0.018 | 102 | 3 | 108 |
| **5** | T1 | 1 | 13 | 1999 | cf EBRT | 3D-CRT | 68 | 0 | - | 6 | TAB | <0.1 | 168 | 18 | 231 |
| **6** | T1 | 1 | 10 | 2008 | cf EBRT | IMRT | 72 | 3 | Gold | No ADT | - | 0.75 | 120 | 11 | 132 |
| **7** | T1 | 2 | 14 | 2008 | cf EBRT | IMRT | 76 | 3 | Gold | 6 | LHRH-agonist | <0.4 | 98 | 6 | 122 |
| **8** | T1 | 1 | 9 | 2015 | HDR | HDR | 27 | 0 | - | No ADT | - | 2.8 | 18 | 10 | 45 |
| **9** | T1 | 4 | 37 | 2004 | cf EBRT/sHDRb^b^ | IMRT/HDRb | 72/27 | 3 | Gold | 36 | Bicalutamide | 1.6 | 43 | 24 | 169 |
| **10** | T1 | 1 | 13 | 2007 | cf EBRT | 3D-CRT | 72 | 0 | - | No ADT | - | 1.8 | 48 | 10 | 134 |
| **11** | T3 | 3 | 22 | 2010 | cf EBRT | IMRT | 72 | 3 | Gold | 36 | Bicalutamide | 0.14 | 84 | 3 | 103 |
| **14** | T3 | 1 | 47 | 2008 | cf EBRT | IMRT | 74 | 3 | Gold | 24 | LHRH-agonist | 0.2 | 120 | 4 | 132 |
| **15** | T2 | 3 | 23 | 2004 | cf EBRT | 3D-CRT | 70 | 0 | - | No ADT | - | 1.1 | 182 | 5 | 186 |
| **16** | T4 | 1 | 29 | 2000 | cf EBRT | 3D-CRT | 70 | 0 | - | 12 | LHRH-agonist | 0.82 | 53 | 28 | 225 |
| **17** | T3 | 2 | 23 | 2007 | cf EBRT | 3D-CRT | 72 | 0 | - | 24 | LHRH-agonist | 0.045 | 150 | 3 | 155 |
| **18** | T3 | 5 | 15 | 2011 | cf EBRT | IMRT | 76 | 3 | Gold | Continuous | LHRH-agonist | <0.1 | NA | 2 | 100 |
| **19** | T2 | 1 | 30 | 2003 | cf EBRT | 3D-CRT | 72 | 0 | - | 6 | TAB | <0.5 | 26 | 3 | 210 |
| **20** | T1 | 1 | 10 | 2014 | cf EBRT | IMRT | 76 | 3 | Gold | No ADT | - | 1.1 | 64 | 13 | 68 |
| **21** | T2 | 2 | 8 | 2012 | cf EBRT | IMRT | 76 | 3 | Gold | No ADT | - | 0.98 | 91 | 3 | 95 |
| **22** | T3 | 5 | 18 | 2014 | cf EBRT | IMRT | 78 | 3 | Gold | 24 | LHRH-agonist | <0.2 | 63 | 4 | 65 |
| **23** | T3 | 5 | 22 | 2004 | cf EBRT | 3D-CRT | 70 | 0 | - | 24 | LHRH-agonist | 0.18 | 179 | 7 | 192 |
| **24** | T1 | 1 | 5 | 2003 | cf EBRT | 3D-CRT | 70 | 0 | - | No ADT | - | 0.8 | 188 | 7 | 205 |
| **25** | T3 | 2 | 13 | 2008 | cf EBRT | IMRT | 76 | 3 | Gold | 6 | LHRH-agonist | <0.4 | 138 | 3 | 142 |
| **26** | T1 | 4 | 7 | 2013 | hf EBRT | IMRT | 62 | 3 | Gold | No ADT | - | 0.15 | 84 | 3 | 90 |
| **27** | T1 | 1 | 27 | 2002 | cf EBRT | 3D-CRT | 68 | 0 | - | 67 | LHRH-agonist | <0.1 | 96 | 7 | 169 |
| **28** | T2 | 2 | 6 | 2018 | cf EBRT | IMRT | 78 | 3 | Gold | No ADT | - | 0.66 | 31 | 6 | 35 |
| **29** | T1 | 1 | 8 | 2007 | cf EBRT | IMRT | 76 | 3 | Gold | 6 | LHRH-agonist | 0.7 | 31 | 3 | 161 |
| **30** | T2 | 2 | 8 | 2009 | cf EBRT | IMRT | 79 | 3 | Gold | No ADT | - | 0.26 | 138 | 3 | 143 |
| **32** | T1 | 1 | 13 | 2010 | cf EBRT | IMRT | 74 | 3 | Gold | No ADT | - | 1.2 | 56 | 8 | 138 |
| **33** | T1 | 2 | 15 | 2009 | cf EBRT | IMRT | 76 | 3 | Gold | 6 | LHRH-agonist | 0.4 | 73 | 14 | 148 |
| **34** | T1 | 1 | 23 | 2009 | cf EBRT | 3D-CRT | 72 | 0 | - | 6 | LHRH-agonist | 0.093 | 142 | 3 | 148 |
| **36** | T1 | 1 | 2 | 2010 | cf EBRT/sHDRb^b^ | IMRT/HDRb | 72/24 | 3 | Gold | No ADT | - | 0.7 | 82 | 5 | 140 |
| **37** | T2 | 1 | 6 | 2012 | cf EBRT | IMRT | 74 | 3 | Gold | No ADT | - | 1.0 | 38 | 3 | 113 |
| **38** | T2 | 5 | 11 | 2011 | cf EBRT | IMRT | 74 | 3 | Gold | 36 | Bicalutamide | 0.006 | 129 | 3 | 136 |
| **39** | T1 | 1 | 14 | 2013 | cf EBRT | IMRT | 76 | 3 | Gold | No ADT | - | 0.38 | 94 | 8 | 108 |
| **40** | T2 | 2 | 10 | 2015 | hf EBRT | VMAT | 60 | 3 | Nitinol | 36 | LHRH-agonist | 0.039 | 57 | 5 | 80 |
| **41** | T2 | 5 | 25 | 2011 | cf EBRT | IMRT | 77 | 3 | Nitinol | 36 | LHRH-agonist | 0.007 | 127 | 2.3 | 141 |
| **42** | T3 | 2 | 19 | 2016 | hf EBRT | IMRT | 60 | 3 | Gold | 12 | LHRH-agonist | 0.5 | 71 | 6 | 77 |
| **43** | T1 | 1 | 10 | 2009 | cf EBRT | IMRT | 76 | 3 | Gold | 9 | LHRH-agonist | <0.1 | 138 | 2 | 155 |

ADT = androgen deprivation therapy; BCR = biochemical recurrence; cf = conventional fractionation; EBRT = external beam RT; hf = hypofractionation; IMRT = intensity-modulated RT; ISUP GG = International Society of Urological Pathology grade group; LHRH = luteinizing hormone-releasing hormone; LN = lymph node; PCa = prostate cancer; PSA = prostate-specific antigen; Pt = patient number; pTx = primary treatment; RT = radiation therapy; sHDRb = salvage high dose rate brachytherapy; sTULSA = salvage magnetic resonance imaging-guided transurethral ultrasound ablation; TAB = total androgen blockage; 3D-CRT = three-dimensional conformal RT; VMAT = volumetric modulated arc therapy

^a^ BCR after primary RT was defined as a PSA rise above the nadir of more than 2 ng/ml.

^b^ Pts 9 and 36 received sHDRb (3 x 9 Gy without ADT and 3 x 8 Gy combined with 6-month degarelix) in 2011 and 2018 for histologically verified radiorecurrent PCa after primary EBRT.

Additional information:

* Pt 2 had a right-sided salvage robot-assisted laparoscopic iliac lymphadenectomy for solitary PSMA PET-positive LN disease in 2018. Pathology showed 3 of 9 LNs with PCa metastasis.

* Pt 27 initially received ADT monotherapy for metastatic PCa based on a bone scan. Follow-up imaging suggested localized disease, leading to definitive RT in 2007. ADT continued for 6 mo and was then discontinued after undetectable PSA levels.

* Pt 30´s open radical prostatectomy was aborted due to technical difficulties, but bilateral iliac LN dissection was performed in 2009, with no PCa detected on pathology (0/10 LNs). Later the same year, the pt underwent EBRT.

# **Supplementary Table 5. Treatment parameters and perioperative outcomes related to salvage MRI-guided transurethral ultrasound ablation**

| **Pt** | **Ablation pattern** | **Treatment coverage (%TPV)** | **Sonication time** | **No of sonication** | **Postoperative catheterization** | **Duration of catheterization (d)** | **Hospitalization**  **(d)** |
| --- | --- | --- | --- | --- | --- | --- | --- |
| **1** | Subtotal, posterobasal region untreated | 75 | 44 | 2 | Transurethral | 1 | 1 |
| **2** | Right apex to midgland quadrant | 25 | 22 | 2 | Transurethral | 1 | 1 |
| **3** | Whole gland | 100 | 39 | 2 | Transurethral | 14 | 1 |
| **4** | RL hemiablation | 50 | 50 | 2 | Transurethral | 7 | 1 |
| **5** | LL hemiablation | 50 | 55 | 2 | Transurethral | 1 | 1 |
| **6** | RL hemiablation | 50 | 52 | 3 | Transurethral | 7 | 1 |
| **7** | Anterior and LL hemiablation | 75 | 50 | 2 | Transurethral | 1 | 1 |
| **8** | RL hemiablation | 50 | 22 | 2 | Transurethral | 1 | 1 |
| **9** | Whole gland | 100 | 50 | 2 | Transurethral | 14 | 1 |
| **10** | Anterior and LL hemiablation | 75 | 49 | 3 | SPC | 36 | 1 |
| **11** | Whole gland | 100 | 67 | 5 | SPC | 23 | 1 |
| **14** | Anterior hemiablation | 50 | 48 | 3 | SPC | 34 | 1 |
| **15** | Whole gland | 100 | 24 | 2 | SPC | 13 | 1 |
| **16** | LL hemiablation | 50 | 45 | 2 | SPC | 20 | 1 |
| **17** | Whole gland | 100 | 35 | 2 | SPC | 20 | 1 |
| **18** | Whole gland | 100 | 75 | 2 | SPC | Permanent | 2 |
| **19** | Whole gland | 100 | 54 | 2 | SPC | Permanent | 1 |
| **20** | Whole gland | 100 | 36 | 2 | SPC | 41 | 1 |
| **21** | Whole gland | 100 | 39 | 2 | SPC | 13 | 1 |
| **22** | Whole gland | 100 | 47 | 2 | SPC | 18 | 1 |
| **23** | Whole gland | 100 | 48 | 2 | SPC | 16 | 1 |
| **24** | Whole gland | 100 | 73 | 2 | SPC | 97 | 1 |
| **25** | Whole gland | 100 | 71 | 2 | SPC | 14 | 1 |
| **26** | Whole gland | 100 | 122 | 2 | SPC | 28 | 1 |
| **27** | Whole gland | 100 | 39 | 2 | SPC | 21 | 1 |
| **28** | Whole gland | 100 | 47 | 2 | SPC | 21 | 1 |
| **29** | Whole gland | 100 | 63 | 2 | SPC | 69 | 1 |
| **30** | Whole gland | 100 | 52 | 2 | Transurethral | 30 | 1 |
| **32** | Whole gland | 100 | 59 | 2 | SPC | 44 | 1 |
| **33** | Whole gland | 100 | 20 | 2 | SPC | 28 | 1 |
| **34** | Anterior hemiablation | 50 | 18 | 2 | SPC | 13 | 1 |
| **36** | Whole gland | 100 | 18 | 2 | SPC | 30 | 1 |
| **37** | Whole gland | 100 | 40 | 2 | SPC | 23 | 1 |
| **38** | Whole gland | 100 | 28 | 2 | SPC | 15 | 1 |
| **39** | Whole gland | 100 | 28 | 1 | SPC | 26 | 1 |
| **40** | RL hemiablation | 50 | 48 | 2 | SPC | 14 | 1 |
| **41** | Whole gland | 100 | 27 | 2 | SPC | 14 | 1 |
| **42** | LL hemiablation | 50 | 11 | 2 | SPC | 15 | 1 |
| **43** | RL hemiablation | 50 | 40 | 2 | SPC | 28 | 1 |

LL = left lobe; NA = not available; Pt = patient number; RL = right lobe; SPC = suprapubic catheter.

**Supplementary Table 6. Adverse events after salvage MRI-guided transurethral ultrasound ablation: Bolded events attributable to the study intervention**

| **Pt** | **Date of sTULSA**  **(mo/yr)** | **No of AEs** | **Postoperative complications**  **(Clavien Dindo)** | | **Longer-term adverse events**  **(Clavien Dindo)** | | | **Inpatient readmission and date (mo/yr)** | **(Re)operations during follow-up after sTULSA** | | **Possible risk factors for complications** | |
| --- | --- | --- | --- | --- | --- | --- | --- | --- | --- | --- | --- | --- |
|  |  |  | **< 90 d** | | **>90 d** | | |  |  |  | **Urological interventions before sTULSA** | **Baseline cystoscopy^1^** |
|  |  |  | **Type and date (mo/yr)** | **Grade** | **Type and date (mo/yr)** | **Grade** | |  | **Procedure (mo/yr)** | **Cause** |  |  |
| **1** | **4/18** | 1 | - | - | Ischemic heart disease (6/21) | 3a | | Ischemic heart disease (6/21) | PTCA (6/21) | Ischemic heart disease | Prostate Bx x2  FM-guided EBRT -07 | No strictures |
| **2** | **7/18** | 1 | - | - | Spondylodiscitis (11/18) | 2 | | Spondylodiscitis (11/18) | - | - | Prostate Bx x2  EBRT -05  Laparoscopic sLND dx -18 | No strictures |
| **3** | **7/18** | 3 | **UR/UTI (8/18)** | **3a** | **UTI (12/18)** | **2** | | **UR/UTI (8/18)** | **2J-ureterstents/ SPC (8/18)** | UR/UTI/suspicion of hydronephrosis | Prostate Bx x2  EBRT -07 | Bulbar urethral stricture |
|  |  |  |  |  | **UTI (8/19)** | **2** | |  |  |  |  |  |
| **4** | **11/18** | 0 | - | - | - | - | | - | - | - | Prostate Bx x3  EBRT -09 | Bulbar urethral stricture |
| **5** | **1/19** | 0 | - | - | - | - | | - | - | - | Prostate Bx x8  EBRT -99  Urethral dilation -19 | Bulbar urethral stricture |
| **6** | **1/19** | 1 | - | - | **UTI** **(9/19)** | **2** | | **UTI (9/19)** | **Cystoscopy without anesthesia (9/19)** | Removal of dystrophic calcification | Prostate Bx x2  TURP -99  FM-guided EBRT -08 | No strictures |
|  |  |  |  |  | **Dystrophic calcification (9/19)** | **2** | |  |  |  |  |  |
| **7** | **3/19** | 1 | **UR/UTI (3/19)** | **2** | - | - | | - | - | - | Prostate Bx x2  FM-guided EBRT -08 | No strictures |
| **8** | **3/19** | 0 | - | - | - | - | | - | - | - | Prostate Bx x2  HDRb -15 | No strictures |
| **9** | **4/19** | 2 | **UR/UTI (5/19)** | **2** | **Bulbar urethral stricture (7/22)** | **3b** | | **UR/UTI (5/19)** | **Optic urethrotomy/SPC (7/22)** | Exacerbated bulbar urethral stricture | Prostate Bx x3  FM-guided EBRT -05  sHDRb -11 | Bulbar urethral stricture |
|  |  |  |  |  |  |  |  |  | **Cystectomy with Bricker diversion (1/23)** | Bladder/prostatic cavity stones with no bladder access due to urethral stricture |  |  |
| **10** | **5/19** | 1 | **UR (6/19)** | **1** | - | - | | - | - | - | Prostate Bx x 5  TURP -07  EBRT -08 | No strictures |
| **11** | **6/19** | 2 | **UTI (9/19)** | **2** | **Puboprostatic fistula (12/23)** | **2** | | **Puboprostatic fistula (12/23)** | - | - | Prostate Bx x2  FM-guided EBRT -10 | No strictures |
| **14** | **11/19** | 1 | - | - | Stroke (12/22) | 2 | | Stroke (12/22) | - | - | Prostate Bx x2  FM-guided EBRT -08 | No strictures |
| **15** | **1/20** | 3 | **UR/UTI** **(2/20)** | **2** | **Epididymitis** **(4/20)** | **2** | | **Epididymitis** (2/21) | - | - | Prostate Bx x2  EBRT -04 | Bulbar urethral stricture |
|  |  |  |  |  | **Epididymitis (2/21)** | **2** | |  |  |  |  |  |
| **16** | **2/20** | 2 | **UTI (2/20)** | **2** | Supraventricular arrhythmia | 3a | | - | Pacemaker implantation (4/22) | Atrial fibrillation | Prostate Bx x2  EBRT -01 | Bulbar urethral stricture |
| **17** | **3/20** | 1 | **UR/UTI (4/20)** | **2** | - | | - | **UR/UTI** **(4/20)** | - | - | Prostate Bx x2  EBRT -07 | No strictures |
| **18** | **3/20** | 3 | **Pelvic pain (3/20)** | **1** | **Puboprostatic fistula** **(7/20)** | **2** | | **Puboprostatic fistula (7/20)** | - | - | Prostate Bx x4  FM-guided EBRT -11 | Bulbar urethral stricture |
|  |  |  | **UR/UTI (5/20)** | **2** |  |  |  |  |  |  |  |  |
| **19** | **10/20** | 1 | **Pelvic pain (10/20)** | **1** | - | - | | - | - | - | Prostate Bx x2  EBRT -03  Optic urethrotomy x2 -20 | Bulbar urethral strictures |
| **20** | **9/20** | 1 | **UTI (10/20)** | **2** | - | - | | - | - | - | Prostate Bx x4  FM-guided EBRT -14 | No strictures |
| **21** | **10/20** | 0 | - | - | - | - | | - | - | - | Prostate Bx x2  FM-guided EBRT -12 | No strictures |
| **22** | **9/20** | 0 | - | - | - | - | | - | - | - | Prostate Bx x2  FM-guided EBRT -15  TURP -16  Cystolithotomy (2cm) -20 | No strictures |
| **23** | **11/20** | 2 | - | - | **Bulbar urethral stricture** **(6/21)** | **3b** | | Ischemic heart disease (8/21) | **Optic urethrotomy** **(8/21)** | Exacerbated bulbar urethral stricture | Prostate Bx x2  EBRT -04  TURB -20 | Bulbar urethral stricture |
|  |  |  |  |  | Ischemic heart disease (8/21) | 2 | |  |  |  |  |  |
| **24** | **12/20** | 2 | **UR/UTI (12/20)** | **2** | **UR/UTI** **(3/21)** | **2** | | **UR/UTI** **(12/20)** | - | - | Prostate Bx x3  EBRT -03 | Bulbar urethral stricture |
| **25** | **3/21** | 0 | **-** | **-** | - | - | | - | - | - | Prostate Bx x2  FM-guided EBRT -09 | Bulbar urethral stricture |
| **26** | **4/21** | 0 | **-** | **-** | - | - | | - | - | - | Prostate Bx x2  FM-guided EBRT -13 | No strictures |
| **27** | **5/21** | 0 | **-** | **-** | - | - | | - | - | - | Prostate Bx x5  EBRT -07 | No strictures |
| **28** | **5/21** | 4 | **Pelvic pain (6/21)** | **1** | **Puboprostatic fistula** (4/23) | **3b** | | **UR/UTI** **(7/21)** | **Cystectomy with**  **Bricker diversion (10/23)** | Puboprostatic fistula/osteitis pubis | Prostate Bx x2  FM-guided EBRT -18 | No strictures |
|  |  |  | **UR/UTI (7/21)** | **2** | Pulmonary embolism (1/24) | 4b | | Pulmonary embolism (1/24) |  |  |  |  |
| **29** | **7/21** | 1 | **UR** | **1** | - | - | | - | - | - | Prostate Bx x2  FM-guided EBRT -08 | Bulbar urethral stricture |
| **30** | **10/21** | 0 | **-** | **-** | - | - | | - | - | - | Prostate Bx x2  Open RP trial, LND l.a -09  FM-guided EBRT -09 | No strictures |
| **32** | **1/22** | 3 | **UTI (2/22)** | **2** | **UTI (4/22)** | **2** | | **UTI** **(2/22)** | - | - | Prostate Bx x6  TURP -09  FM-guided EBRT -10 | Bulbar urethral stricture |
|  |  |  |  |  | **Epididymitis** **(6/22)** | **2** | | **UTI (4/22)** |  |  |  |  |
|  |  |  |  |  |  |  |  | **Epididymitis (6/22)** |  |  |  |  |
| **33** | **4/22** | 0 | - | - | - | - | | - | - | - | Prostate Bx x4  FM-guided EBRT -09 | Bulbar urethral stricture |
| **34** | **5/22** | 0 | - | - | - | - | | - | - | - | Prostate Bx x3  EBRT -09 | No strictures |
| **36** | **8/22** | 3 | - | - | **UTI (12/22)** | **2** | | - | - | - | Prostate Bx x3  TURP -10  FM-guided EBRT -10  sHDRb -18  Optic urethrotomy -22 | Bulbar urethral stricture |
|  |  |  |  |  | **UTI (10/23)** | **2** | |  |  |  |  |  |
|  |  |  |  |  | **UR (12/23)** | **2** | |  |  |  |  |  |
| **37** | **11/22** | 0 | - | - | - | - | | - | - | - | Prostate Bx x2  FM-guided EBRT -13 | Bulbar urethral stricture |
| **38** | **12/22** | 0 | - | - | - | - | | - | - | - | Prostate Bx x2  FM-guided EBRT -11 | No strictures |
| **39** | **1/23** | 5 | **UR/UTI (3/23)** | **2** | **UR/UTI (6/23)** | **2** | | Ischemic heart disease (1/24) | - | - | Prostate Bx x2  FM-guided EBRT -13  TURP -22 | Bulbar urethral stricture |
|  |  |  |  |  | **UR/UTI (8/23)** | **2** | |  |  |  |  |  |
|  |  |  |  |  | **UR/UTI (10/23)** | **2** | |  |  |  |  |  |
|  |  |  |  |  | Ischemic heart disease (1/24) | 2 | |  |  |  |  |  |
| **40** | **2/23** | 1 | **UTI (4/23)** | **2** | - | - | | - | - | - | Prostate Bx x2  TURP -11  FM-guided EBRT -16 | No strictures |
| **41** | **2/23** | 0 | **-** | **-** | - | - | | - | - | - | Prostate Bx x2  FM-guided EBRT -11 | No strictures |
| **42** | **3/23** | 1 | **UTI (4/23)** | **2** | - | - | | - | - | - | Prostate Bx x2  FM-guided EBRT -16 | No strictures |
| **43** | **3/23** | 1 | **UR/UTI (5/23)** | **2** | - | - | | - | - | - | Prostate Bx x3  FM-guided EBRT -10  TURP -22 | Bulbar urethral stricture |

AE = adverse event; Bx = prostate biopsy; EBRT = external beam radiotherapy; FM = fiducial marker; sLND = salvage lymph node dissection; HDRb = high dose rate brachytherapy; Pt = patient number; PTCA = percutaneous transluminal coronary angioplasty; RP = radical retropubic prostatectomy; SPC = suprapubic catheter; sTULSA = salvage MRI-guided transurethral ultrasound ablation; TUIP = transurethral incision of the prostate; TURB = transurethral resection of the bladder; TURP = transurethral resection of the prostate; UR = urinary retention; UTI = urinary tract infection

^1^ Every patient underwent outpatient flexible cystoscopy at baseline to confirm urethral patency for ultrasound applicator insertion, and all showed typical signs of radiation cystitis. Baseline cystoscopy revealed a relative bulbar urethral stricture (visible but accessible bladder) in pts 3, 4, 9, 15, 16, 18, 23-25, 29, 32, 33, 37, 39, and 43. Pts 5, 19, and 36 had no bladder access due to urethral stricture and required optic urethrotomy and urethral dilation before sTULSA.

**Supplementary Table 7. Comparison of severe genitourinary toxicity based on ablation pattern (whole-gland vs. partial gland ablation)**


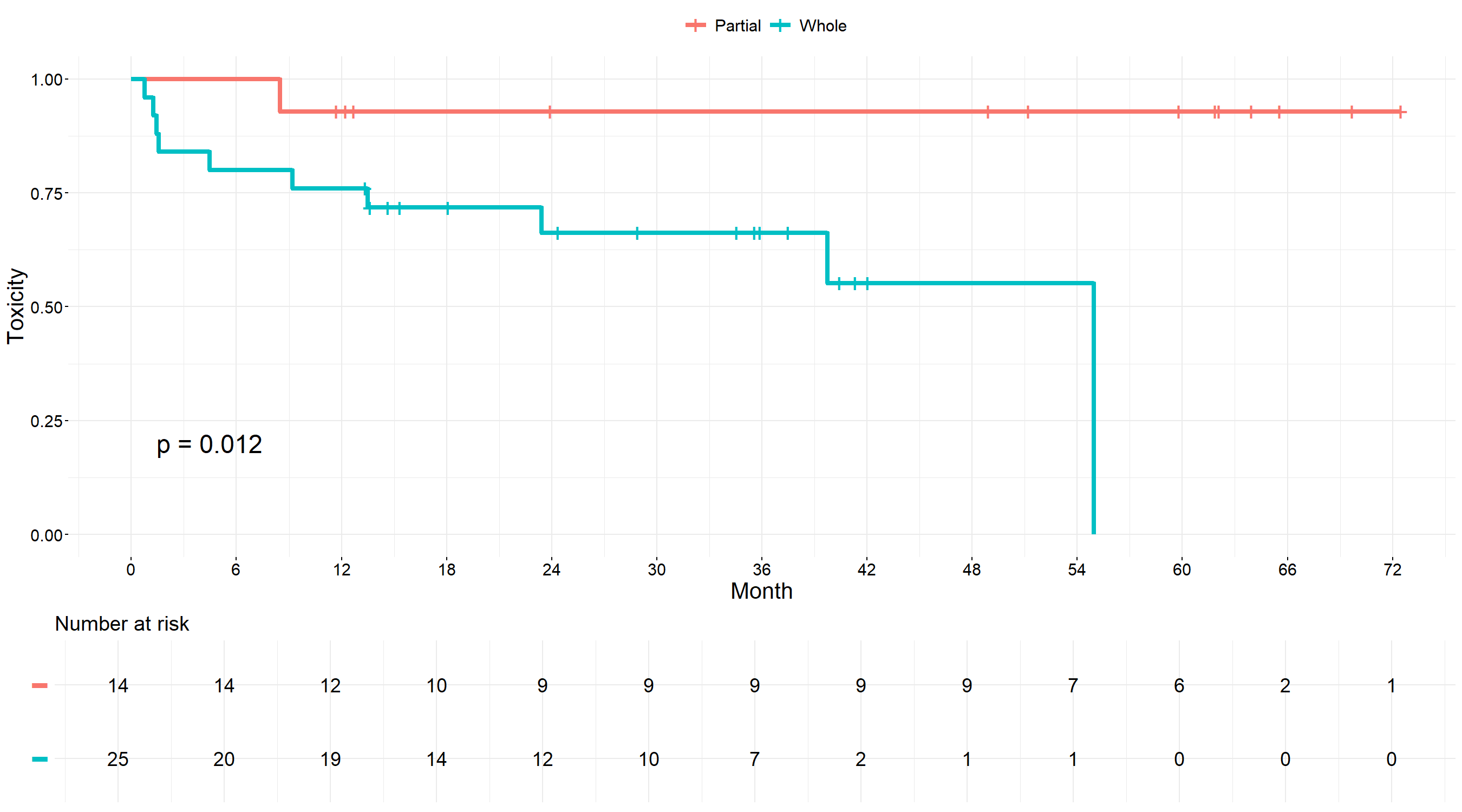


The primary safety endpoint was the occurrence of severe genitourinary and gastrointestinal toxicities, defined as any Clavien-Dindo ≥3 event or toxicity requiring hospitalization.

# **Supplementary Table 8. Association between the ablation pattern (whole-gland vs. partial gland ablation) and the number of adverse events**

| **Characteristic** | **Ablation pattern** | | | **p-value**^2^ |
| --- | --- | --- | --- | --- |
|  | **Partial gland**^1^ (n=14) | **Whole gland**^1^ (n=25) | **Overall**^1^ (n=39) |  |
| **Number of adverse events** | 0.50 (0.00, 1.00) | 1.00 (0.00, 3.00) | 1.00 (0.00, 1.50) | 0.11 |
| **Adverse event** |  |  |  | 0.5 |
| **No** | 7 (50%) | 10 (40%) | 17 (44%) |  |
| **Yes** | 7 (50%) | 15 (60%) | 22 (56%) |  |
| ^1^Median (IQR); n(%) | | | |  |
| ^2^Wilcoxon rank sum test; Pearson´s Chi-squared test | | | | |

There was no statistically significant difference in the number of adverse events between whole-gland and partial gland ablation.

# **Supplementary Table 9. Uroflowmetry outcomes during 12 months follow-up**

| Pt | **Baseline** | | | | **3 mo** | | | | **6 mo** | | | | **12 mo** | | | |
| --- | --- | --- | --- | --- | --- | --- | --- | --- | --- | --- | --- | --- | --- | --- | --- | --- |
|  | Voided volume (ml) | Qmax (ml/s) | Qave (ml/s) | PVR (ml) | Voided volume (ml) | Qmax (ml/s) | Qave (ml/s) | PVR (ml) | Voided volume (ml) | Qmax (ml/s) | Qave (ml/s) | PVR (ml) | Voided volume (ml) | Qmax (ml/s) | Qave (ml/s) | PVR (ml) |
| 1 | 431 | 13.9 | 6.8 | 166 | 446 | 12.5 | 5.6 | 58 | 163 | 8.1 | 4.2 | 0 | 174 | 10.5 | 5.4 | 0 |
| 2 | 188 | 11.3 | 4.2 | 0 | 92 | 5.8 | 2.8 | 39 | NA | NA | NA | NA | 186.3 | 10.8 | 5.4 | 13 |
| 3 | 285 | 7.1 | 3.1 | 101 | 255 | 4.0 | 1.5 | 49 | 139 | 5.0 | 2.1 | 30 | 199 | 6.2 | 4.1 | 0 |
| 4 | 465 | 11.2 | 5.6 | 49 | 64 | 3.5 | 1.6 | 0 | 183 | 6.7 | 2.8 | 8 | 284 | 9.5 | 3.8 | 90 |
| 5 | 275 | 11.4 | 7.6 | 0 | 154 | 10.1 | 4.2 | 0 | 144 | 11.4 | 5.9 | 0 | 154 | 10.5 | 4.5 | 0 |
| 6 | 256 | 25.7 | 13 | 0 | 78 | 10.9 | 6.1 | 0 | 40 | 4.7 | 2.4 | 0 | 63 | 3.3 | 1.7 | 0 |
| 7 | 735 | 17.2 | 9.2 | 146 | 233 | 4.0 | 2.3 | 220 | 183 | 10.2 | 5.8 | 65 | 264 | 8.3 | 4.9 | 186 |
| 8 | 615 | 13.5 | 7.1 | 64 | 283 | 16.3 | 8.6 | 35 | 220 | 17.6 | 8.1 | 0 | 340 | 21 | 11.5 | 20 |
| 9 | 433 | 7.9 | 4.0 | 143 | SPC | | | | SPC | | | | 48 | 1.7 | 0.8 | 250 |
| 10 | 405 | 20.8 | 9.9 | 100 | 257 | 16.7 | 8.0 | 42 | 191 | 14.4 | 6.0 | 40 | 224 | 15.3 | 7.1 | 72 |
| 11 | 147 | 15.1 | 4.2 | 0 | 70 | 7.0 | 2.6 | 0 | 76 | 6.5 | 3.2 | 0 | 81 | 6.1 | 3.2 | 19 |
| 14 | 412 | 15 | 8.6 | 0 | 261 | 5.5 | 2.4 | 36 | NA | NA | NA | NA | 160 | 6.5 | 4.2 | 20 |
| 15 | 264 | 9.8 | 4.2 | 443 | 131 | 7.7 | 3.8 | 101 | 422.4 | 9.1 | 6.0 | 129 | 296.7 | 11.4 | 4.4 | 150 |
| 16 | 66 | 7.6 | 3.7 | 91 | 163 | 6.6 | 2.6 | 89 | 204 | 10.4 | 5.1 | 22 | 177 | 9.3 | 5.5 | 56 |
| 17 | 132 | 9.7 | 4.7 | 30 | Withdrew from the follow-up | | | | | | | | | | | |
| 18 | 138 | 11.7 | 3.4 | 156 | SPC | | | | SPC | | | | SPC | | | |
| 19 | 58 | 1.7 | 0.8 | 0 | SPC | | | | SPC | | | | SPC | | | |
| 20 | 237 | 12.7 | 4.8 | 0 | 74.3 | 3.7 | 1.7 | 84 | NA | NA | NA | NA | 105.6 | 3.9 | 1.7 | 24 |
| 21 | 251 | 26.3 | 8.7 | 29 | 213 | 14 | 6.6 | 18 | 230 | 13.0 | 6.8 | 43 | 218 | 11.1 | 6.2 | 9 |
| 22 | 196 | 12.1 | 3.7 | 7 | 161 | 7.8 | 4.6 | 69 | 218 | 8.8 | 4.7 | 141 | 209 | 9.4 | 4.2 | 86 |
| 23 | 176 | 13.7 | 7.8 | 25 | 153 | 5.4 | 2.5 | 139 | 160 | 7.6 | 2.7 | 190 | NA | NA | NA | 28 |
| 24 | 327.8 | 13.6 | 7.2 | 149 | 145 | 6.6 | 3.3 | 49 | 111 | 6.6 | 3.3 | 53 | 45 | 11.7 | 3.8 | 0 |
| 25 | 124 | 15 | 7.3 | 10 | NA | NA | NA | 20 | 95 | NA | NA | 34 | 80 | 11.4 | 3.8 | 9 |
| 26 | 182.3 | 15.4 | 8.4 | 54 | 45 | 2.5 | 1.2 | 54 | 94.5 | 7.5 | 3.0 | 15 | 37 | 3.9 | 1.9 | 8 |
| 27 | 168 | 17.4 | 8.8 | 34 | 161 | 9.0 | 3.5 | 37 | 91.8 | 8.4 | 3.3 | 23 | 361 | 14.3 | 6.4 | 85 |
| 28 | 880 | 38.1 | 18.1 | 18 | 152 | 9.6 | 3.9 | 47 | 243.2 | 14.7 | 6.0 | 11 | 157 | 5.8 | 3.1 | 94 |
| 29 | 278 | 20 | 9.8 | 51 | 91 | 5.8 | 2.1 | 15 | 63.8 | 4.4 | 1.8 | 100 | 69 | 3.8 | 1.4 | 22 |
| 30 | 111 | 11.7 | 5.2 | 32 | 43.9 | 15.5 | 3.8 | 0 | 40.1 | 6.4 | 2.5 | 44 | 116 | 14.3 | 7.1 | 0 |
| 32 | 255.4 | 17.4 | 7.7 | 0 | 38.9 | 2.4 | 1.1 | 30 | NA | NA | NA | NA | 208.5 | 10.7 | 5.2 | 0 |
| 33 | 148.1 | 9.9 | 4.3 | 86 | 129 | 7.4 | 3.9 | 74 | 148.6 | 11.3 | 5.2 | 233 | 188.5 | 10.5 | 4.9 | 116 |
| 34 | 424.7 | 21.3 | 10.6 | 70 | 292.7 | 17.3 | 8.6 | 0 | 365.7 | 25 | 11.8 | 41 | 474.7 | 19 | 11.5 | 50 |
| 36 | 293.3 | 5.6 | 2.8 | 150 | 60.2 | 5.6 | 2.6 | 0 | 224.6 | 5.6 | 3.3 | 132 | 188.4 | 10.7 | 5.8 | 23 |
| 37 | 907.3 | 17.6 | 7.7 | 7 | 322.3 | 11.4 | 5.1 | 133 | 115 | 6.3 | 3.3 | 0 | 340.4 | 20.1 | 10.4 | 7 |
| 38 | 105 | 10.9 | 5.5 | 0 | 103.9 | 9.4 | 4.4 | 0 | 77.9 | 8.2 | 4.1 | 12 | 75 | 10.3 | 4.7 | 0 |
| 39 | 300.2 | 11.3 | 3.6 | 3 | 100.6 | 4.0 | 1.0 | 301 | 61.5 | 1.1 | 0.6 | 225 | Transurethral catheter | | | |
| 40 | 595 | 25.3 | 16.5 | 200 | 349.4 | 11.9 | 7.0 | 270 | 191.3 | 7.6 | 5.9 | 142 | 858.2 | 22.7 | 14.5 | 159 |
| 41 | 206 | 10.5 | 5.8 | 48 | 173.4 | 12.4 | 6.3 | 25 | 242.7 | 16.9 | 8.7 | 17 | 283.4 | 15.6 | 9.4 | 80 |
| 42 | 164.2 | 14.4 | 5.6 | 14 | 98.5 | 9.3 | 3.8 | 24 | 61.3 | 8.3 | 2.7 | 15 | 138.6 | 9.7 | 4.7 | 27 |
| 43 | 266.1 | 15.7 | 5.7 | 23 | Transurethral catheter | | | | SIC | | | | 175.2 | 3.9 | 1.6 | 47 |

NA = not available; Pt = patient number; PVR = post-void residual volume; SPC = suprapubic catheter; SIC = self-intermittent catheterization; Qave = average urinary flow rate; Qmax = maximum urinary flow rate

* Pt 5, 19 and 36 had bulbar urethral strictures identified during the baseline cystoscopy, which were treated with optical urethrotomy prior to undergoing sTULSA.

* Pt 39, 43 on transurethral catheter. Catheter removal trials unsuccessful.

# **Supplementary Table 10. Oncological outcomes at 12 months after salvage MRI-guided transurethral ultrasound ablation.**

| **Pt** | **Biopsy status^a^** | | | | | **Imaging status** | | | | **PSA (ng/ml)** | | |
| --- | --- | --- | --- | --- | --- | --- | --- | --- | --- | --- | --- | --- |
|  | **Positive cores/total cores** | | **Total length (mm)** | | **ISUP GG^d^** |  |  |  |  | **Baseline** | **Nadir** | **1 yr after sTULSA** |
|  | **In-field** | **Out-of-field** | **Bx** | **PCa** |  | **Prostate volume on MRI (cc)** | **Prostate and SV** | | **mi+rTNM status** |  |  |  |
|  |  |  |  |  |  |  | **mpMRI**  **(PI-RR)^e^** | **PSMA PET (SUVmax g/ml)** |  |  |  |  |
| **1*** | 0/4 | 1/4 | 87 | 1.0 | 4 | 10 | Negative | Right SV (4.8) | T3bN0M0 | 1.9^b+c^ | 0.12 | 0.65 |
| **2** | 0/4 | - | 60 | - | - | 32 | Negative | Negative | No disease | 5.5 | 1.2 | 1.4 |
| **3** | 0/4 | - | 69 | - | - | 6 | Negative | Negative | No disease | 7.5^b+c^ | 0.006 | 0.2 |
| **4** | 0/4 | - | 48 | - | - | 10 | Negative | Negative | No disease | 3.3 | 0.27 | 0.34 |
| **5** | 1/4 | - | 30 | 1.5 | 2 | 10 | Negative | Left lobe (5.1) | T2N0M0 | 16 | 0.47 | 1.4 |
| **6** | 0/4 | - | 43 | - | - | 18 | Negative | Negative | No disease | 11 | 0.13 | 0.18 |
| **7** | 0/6 | - | 53 | - | - | 12 | Negative | Negative | No disease | 4.7 | 0.19 | 0.24 |
| **8** | 0/5 | - | 75 | - | - | 11 | Negative | Negative | No disease | 0.37^b+c^ | <0.006 | 0.07 |
| **9*** | 0/4 | 1/2 | 75 | 4.0 | 4 | No prostate detectable | Positive (4) | Right SV (6.2) | T3bN0M1a | 13 | 4.7 | 1.1^b^ |
| **10** | 0/6 | - | 90 | - | - | 9 | Negative | Negative | No disease | 9.5 | 0.18 | 0.18 |
| **11** | 0/6 | - | 68 | - | - | 1 | Negative | Negative | No disease | 0.079^b+c^ | 0.037 | 0.22 |
| **14** | 0/2 | 4/4 | 72 | 8 | 2 | 7 | Positive (4) | Negative | T2N0M0 | 3.8 | 0.53 | 0.65 |
| **15** | 0/4 | - | 51 | - | - | 1 | Negative | Negative | No disease | 4.8 | 0.061 | 0.11 |
| **16** | 0/3 | - | 45 | - | - | 5 | Negative | Negative | No disease | 9.2^b+c^ | <0.006 | 0.048 |
| **17** | - | - | - | - | - | - | - | - | - | 2.9 | <0.006 | <0.006 |
| **18** | - | - | - | - | - | No prostate detectable | Negative | Right SV (11.1) | T3bN0M0 | 1.9^b^ | <0.006^b^ | 0.032^b^ |
| **19** | 0/2 | - | 12 | - | - | 1 | Negative | Negative | No disease | 3.3^b+c^ | <0.006 | 0.019 |
| **20** | 0/2 | - | 20 | - | - | 0.4 | Negative | Negative | No disease | 13 | 0.061 | 0.061 |
| **21** | 0/4 | - | 40 | - | - | 15 | Negative | Negative | No disease | 2.9 | 0.008 | 0.008 |
| **22** | 0/4 | - | 33 | - | - | 0.4 | Negative | Negative | No disease | 3.4 | 0.18 | 0.19 |
| **23** | 0/5 | - | 24 | - | - | 1 | Negative | Negative | No disease | 1.6 | <0.006 | <0.006 |
| **24** | 0/4 | - | 50 | - | - | 1 | Negative | Negative | No disease | 6.6 | 0.22 | 0.63 |
| **25** | 1/9 | - | 121 | 2.5 | 2 | 5 | Negative | Negative | No disease | 2.6 | <0.02 | <0.02 |
| **26** | 0/3 | - | 35 | - | - | 7 | Negative | Negative | No disease | 3.0 | 0.047 | 0.078 |
| **27** | 0/3 | - | 46 | - | - | 6 | Negative | Negative | No disease | 6.3^b+c^ | 0.02 | 0.14 |
| **28*** | 0/5 | 0/4 | 74 | - | - | No prostate detectable | Negative | Right SV (11.2) | T3bN1M0 | 5.7 | 0.025 | 0.41 |
| **29** | 0/6 | 0/3 | 98 | - | - | 2 | Positive (5) | Right SV (3.9) | T3bN0M0 | 1.6^b+c^ | <0.1 | 0.17 |
| **30** | 2/14 | - | 153 | 5.3 | 5 | 17 | Positive (5) | Left lobe (14.5) | T3bN1M0 | 2.8 | 0.45 | 0.89 |
| **32** | 0/6 | - | 74 | - | - | 1 | Negative | Negative | No disease | 3.3^b+c^ | <0.02 | <0.02 |
| **33** | 0/4 | - | 50.5 | - | - | 4 | Negative | Negative | No disease | 2.8^b+c^ | 0.1 | 0.37 |
| **34** | 0/2 | 4/6 | 121.5 | 1.2 | 1 | 12 | Negative | Negative | No disease | 2.9 | 0.17 | 0.23 |
| **36** | 3/12 | - | 109 | 3.5 | 3 | 7 | Negative | Negative | No disease | 2.0 | <0.05 | <0.05 |
| **37** | 0/3 | - | 46.5 | - | - | 0.2 | Negative | Negative | No disease | 0.59^b+c^ | <0.006 | 0.15 |
| **38** | 0/7 | - | 55 | - | - | 12 | Negative | Negative | No disease | 3.3 | <0.006 | <0.006 |
| **39** | 0/4 | - | 40.8 | - | - | 0.5 | Negative | Negative | No disease | 8.1 | <0.006 | 0.17 |
| **40** | 0/6 | - | 63 | - | - | 5 | Negative | Negative | No disease | 4.7^b+c^ | 0.013 | 0.24 |
| **41** | 0/5 | - | 80.3 | - | - | 13 | Negative | Negative | No disease | 0.084^b+c^ | 0.018 | 0.092 |
| **42** | 0/5 | - | 45 | - | - | 26 | Negative | Negative | No disease | 0.46^b+c^ | <0.006 | 0.14 |
| **43** | 0/5 | - | 81.8 | - | - | 10 | Negative | Negative | No disease | 0.84^b+c^ | <0.006 | 0.035 |

ADT = androgen deprivation therapy; BCF = biochemical failure; Bx = biopsy ISUP GG = International Society of Urological Pathology grade group; mi+rTNM = molecular imaging + radiological Tumor Node Metastasis; mpMRI = multiparametric magnetic resonance imaging; NA = not applicable; PCa = prostate cancer; PET = positron emission tomography; PI-RR = Prostate Imaging Recurrence Reporting; PSMA = prostate-specific membrane antigen; PSA = prostate-specific antigen; Pt = patient; sTULSA = salvage MRI-guided transurethral ultrasound ablation; SV = seminal vesicle.

^a^ Biopsy protocol included 2-4 in-field biopsies, with additional out-of-field biopsies taken only if imaging revealed suspicious findings. No routine systematic biopsy was performed as per protocol.

^b^ Pt received ADT.

^c^ ADT was discontinued after sTULSA.

^d^ Pathological determination of ISUP GG for salvage pts is not standardized because of radiation- and ablation-induced changes.

^e^ PI-RR classification is not standardized for grading prostate lesions after salvage ablation therapy.

* Pt 1 underwent focal sTULSA in 2018, but the treatment was incomplete due to technical issues from residual gold fiducial markers. Three mo later, PSMA PET and MRI indicated residual tumor (SUVmax 8.1, PI-RR 5), and the patient received focal re-sTULSA. One yr later, PSMA PET showed out-of-field recurrence in the right SV base, confirmed by biopsy. The pt underwent subtotal re-re-sTULSA treatment in 2019.

* Pt 9 experienced BCF (PSA 7.8) six mo post-sTULSA. PSMA PET and MRI revealed a tumor in the right SV (SUVmax 6.2, PI-RR 4) and a 9 mm para-aortic lymph node (SUVmax 7.1) not visible during screening. The pt received ADT. Imaging remained unchanged at one yr, and SV biopsy confirmed PCa. The pt developed bladder stones and underwent open cystectomy with Bricker diversion in 2023. Final pathology detected PCa at the right ureter orifice.

* Pt 28 had PSMA-positive lesion at the right SV base and an 11 mm iliac lymph node (SUVmax 14.5) one yr post-sTULSA with a PSA of 0.41. Prostate and SV biopsies were negative for cancer. Degarelix and later apalutamide were prescribed for nodal-positive disease, reducing PSA to undetectable levels. Both medications were discontinued after one year due to quality-of-life issues, with PSA remaining undetectable. The patient developed a puboprostatic fistula, treated with open cystoprostatectomy and Bricker diversion two yr post-sTULSA. The PSMA-positive iliac lymph node was also removed. Final pathology showed no vital prostate tissue, and both the SVs and the lymph node were cancer-free, suggesting a false-positive PSMA PET result. At the last follow-up in May 2024, PSA remained undetectable.

**Supplementary Table 11. The evolution of prostate volume and non-perfused volume on mpMRI after salvage MRI-guided transurethral ultrasound ablation**

| **Pt** | **Prostate volume (cc) using elliptic formula** | | | **NPV dimensions (cm)** | | |
| --- | --- | --- | --- | --- | --- | --- |
|  | **Baseline** | **3 mo** | **12 mo** | **Immediate** | **3 mo** | **12 mo** |
| 1 | 18 | 25 | 10 | 2.4 x 3.1 x 2.0 | 3.5 x 4.2 x 3.3 | 1.8 x 1.9 x 1.5 |
| 2 | 37 | 40 | 32 | 2.3 x 1.7 x 2.1 | 2.6 x 1.8 x 2.8 | No NPV visible |
| 3 | 14 | 25 | 6 | 2.5 x 3.0 x 3.0 | 2.8 x 3.9 x 3.2 | No NPV visible |
| 4 | 18 | 18 | 10 | 2.6 x 1.7 x 2.9 | 2.4 x 1.2 x 2.1 | 2.3 x 0.9 x 1.2 |
| 5 | 24 | 20 | 10 | 2.5 x 3.0 x 2.5 | 2.2 x 1.7 x 1.5 | No NPV visible |
| 6 | 21 | 18 | 18 | 2.7 x 3.0 x 2.6 | 2.8 x 2.1 x 2.8 | No NPV visible |
| 7 | 33 | 33 | 12 | 2.8 x 4.2 x 2.3 | 3.2 x 4.9 x 2.8 | No NPV visible |
| 8 | 24 | 18 | 11 | 2.3 x 2.4 x 3.6 | 2.3 x 2.8 x 3.0 | 1.5 x 1.9 x 2.8 |
| 9 | 21 | 19 | 0 (no prostate tissue) | 3.0 x 3.6 x 4.6 | 3.4 x 4.4 x 4.5 | No NPV visible |
| 10 | 20 | NA | 9 | 2.1 x 3.6 x 2.6 | NA | No NPV visible |
| 11 | 16 | NA | 1 | 3.8 x 4.1 x 2.3 | NA | No NPV visible |
| 14 | 20 | 24 | 7 | 3.3 x 1.8 x 3.4 | 2.3 x 4.3 x 2.7 | No NPV visible |
| 15 | 24 | 24 | 1 | 2.4 x 4.5 x 2.6 | 3.0 x 4.9 x 2.6 | No NPV visible |
| 16 | 10 | 9 | 5 | 2.1 x 2.5 x 2.6 | 2.2 x 2.7 x 2.8 | 0.6 x 1.7 x 1.0 |
| 17 | 13 | NA | NA | 2.4 x 3.4 x 2.3 | NA | NA |
| 18 | 30 | 2 | 0 (no prostate tissue) | 2.9 x 4.2 x 3.5 | 2.0 x 1.4 x 1.5 | No NPV visible |
| 19 | 18 | 14 | 1 | 3.9 x 2.5 x 3.0 | 2.7 x 3.8 x 2.6 | No NPV visible |
| 20 | 20 | 23 | 0.4 | 2.0 x 3.3 x 2.5 | 3.1 x 3.9 x 3.6 | No NPV visible |
| 21 | 31 | 23 | 15 | 2.9 x 3.6 x 3.0 | 3.4 x 4.1 x 3.1 | 2.6 x 3.5 |
| 22 | 13 | 19 | 0.4 | 2.6 x 3.8 x 3.9 | 2.7 x 4.2 x 3.6 | No NPV visible |
| 23 | 27 | 14 | 1 | 3.7 x 3.2 x 3,5 | 3.9 x 5.3 x 3.6 | No NPV visible |
| 24 | 23 | 14 | 1 | 2.6 x 3.4 x 3.0 | 2.6 x 3.7 x 2.8 | No NPV visible |
| 25 | 16 | 16 | 5 | 3.4 x 2.6 x 2.8 | 2.4 x 3.3 x 2.8 | 1.7 x 2.8 x 1.4 |
| 26 | 43 | 39 | 7 | 5.0 x 3.7 x 3.8 | 3.7 x 5.6 x 3.6 | No NPV visible |
| 27 | 16 | 10 | 6 | 2.0 x 3.7 x 2.3 | 1.8 x 3.0 x 2.4 | 1.1 x 1.8 x 1.7 |
| 28 | 19 | 18 | 0 (no prostate tissue) | 2.7 x 3.6 x 3.5 | 3.6 x 5.3 x 4.0 | No NPV visible |
| 29 | 19 | 20 | 2 | 2.7 x 3.6 x 2.7 | 3.0 x 4.1 | 0.5 x 0.9 x 1.3 |
| 30 | 19 | 27 | 17 | 2.9 x 3.9 x 3.3 | 3.0 x 4.4 x 3.3 | 2.2 x 3.8 x 2.9 |
| 32 | 21 | 16 | 1 | 2.4 x 3.6 x 3.1 | 2.9 x 4.0 x 2.8 | No NPV visible |
| 33 | 17 | 22 | 4 | 2.2 x 2.7 x 3.0 | 2.8 x 3.2 x 3.1 | 0.4 x 0.6 x 0.8 |
| 34 | 14 | 14 | 12 | 3.0 x 2.1 x 2.1 | 2.0 x 2.9 x 2.0 | 1.8 x 3.1 x 2.2 |
| 36 | 19 | 16 | 7 | 2.4 x 3.2 x 2.5 | 3.1 x 3.9 x 2.9 | 2.2 x 3.1 x 2.2 |
| 37 | 16 | 13 | 0.2 | 2.7 x 3.0 x 2.9 | 1.8 x 3.0 x 2.2 | No NPV visible |
| 38 | 21 | 20 | 12 | 2.4 x 3.8 x 3.1 | 2.9 x 5.4 x 2.7 | 2.0 x 4.0 x 2.2 |
| 39 | 19 | 29 | 0.5 | 3.2 x 4.3 x 3.3 | 3.5 x 4.7 x 3.9 | No NPV visible |
| 40 | 15 | 11 | 5 | 2.3 x 2.5 x 3.0 | 2.2 x 1.8 x 2.7 | No NPV visible |
| 41 | 14 | 23 | 13 | 1.9 x 2.5 x 3.0 | 2.5 x 3.9 x 3.0 | 2.1 x 2.9 x 2.4 |
| 42 | 28 | 26 | 26 | 2.0 x 3.1 x 2.5 | 2.3 x 3.9 x 2.6 | 1.9 x 3.1 x 2.2 |
| 43 | 42 | 50 | 10 | 3.6 x 4.0 x 3.1 | 4.6 x 3.7 x 4.2 | 0.8 x 0.8 x 1.2 |

NA = not available; NPV = non-perfused volume; Pt = patient number

**Supplementary Table 12. Comparison of survival outcomes based on ablation pattern (whole-gland vs. partial gland ablation)**


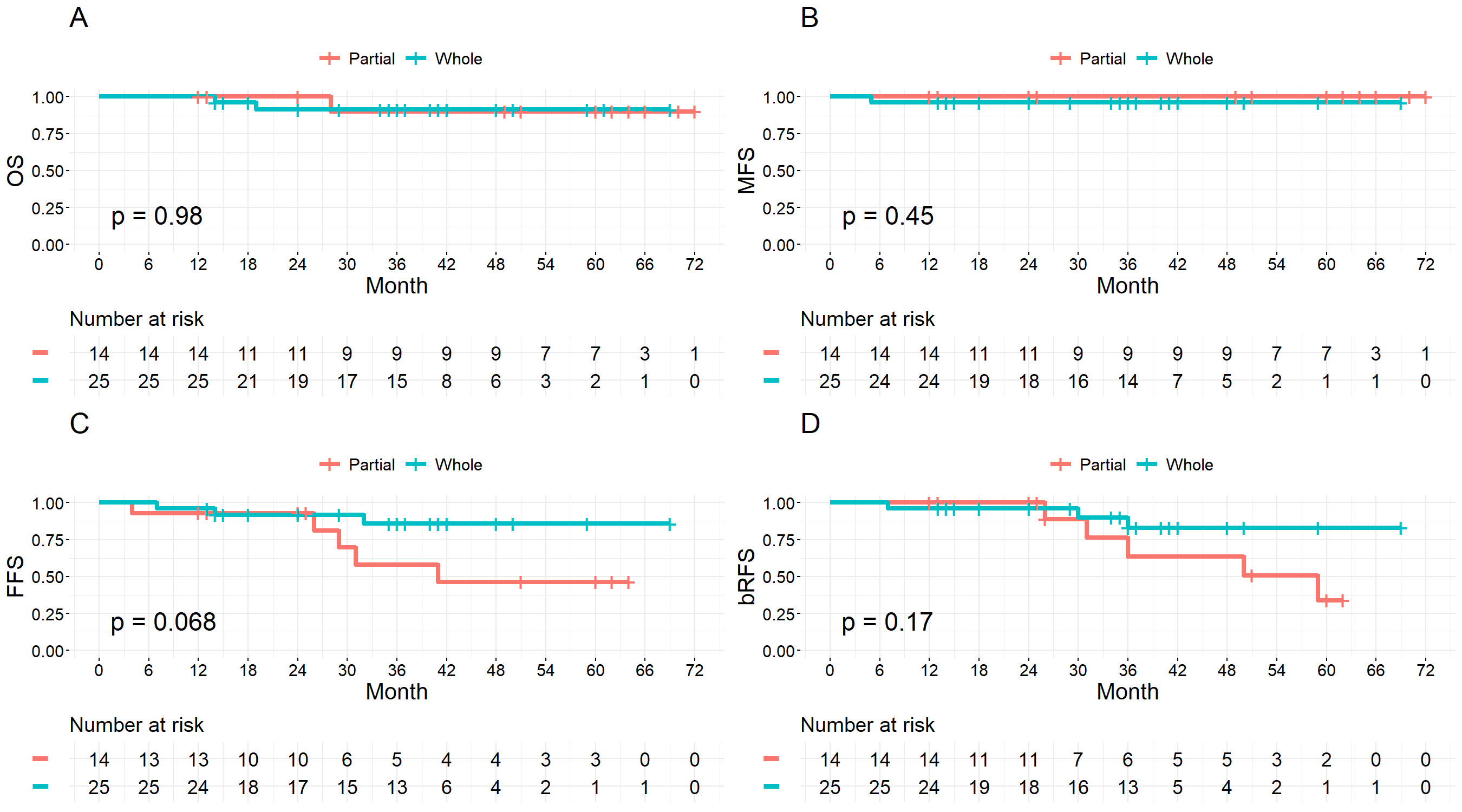


There was no statistically significant difference between WG and partial ablation in any of the survival outcomes.
